# The Rice GLYCINE-RICH PROTEIN 3 Confers Drought Tolerance by Regulating mRNA Stability of ROS Scavenging-Related Genes

**DOI:** 10.1186/s12284-021-00473-0

**Published:** 2021-03-19

**Authors:** Jae Sung Shim, Su-Hyun Park, Dong-Keun Lee, Youn Shic Kim, Soo-Chul Park, Mark Christian Felipe R. Redillas, Jun Sung Seo, Ju-Kon Kim

**Affiliations:** 1 Crop Biotechnology Institute, GreenBio Science and Technology, Seoul National University, Pyeongchang, 25354 South Korea; 2 School of Biological Sciences and Technology, Chonnam National University, Gwangju, 61186 South Korea; 3 Temasek Life Sciences Laboratory, 1 Research Link, National University of Singapore, Singapore, 117604 Singapore; 4 E GREEN GLOBAL, Gunpo, 15843 South Korea; 5 Agriculture and Life Sciences Research Institute, Kangwon National University, Chuncheon, 24341 South Korea; 6 Department of Agricultural Biotechnology, National Academy of Agricultural Science, Rural Development Administration, Jeonju, 54874 South Korea; 7 Biology Department, De La Salle University, 0922 Manila, Philippines

**Keywords:** *OsGRP3*, Drought tolerance, Cytoplasmic foci, RNA-IP, mRNA stability

## Abstract

**Background:**

Plant glycine-rich proteins are categorized into several classes based on their protein structures. The glycine-rich RNA binding proteins (GRPs) are members of class IV subfamily possessing N-terminus RNA-recognition motifs (RRMs) and proposed to be involved in post-transcriptional regulation of its target transcripts. GRPs are involved in developmental process and cellular stress responses, but the molecular mechanisms underlying these regulations are still elusive.

**Results:**

Here, we report the functional characterization of rice *GLYCINE-RICH PROTEIN 3* (*OsGRP3*) and its physiological roles in drought stress response. Both drought stress and ABA induce the expression of *OsGRP3*. Transgenic plants overexpressing *OsGRP3* (*OsGRP3*^*OE*^) exhibited tolerance while knock-down plants (*OsGRP3*^*KD*^) were susceptible to drought compared to the non-transgenic control. In vivo*,* subcellular localization analysis revealed that OsGRP3-GFP was transported from cytoplasm/nucleus into cytoplasmic foci following exposure to ABA and mannitol treatments. Comparative transcriptomic analysis between *OsGRP3*^*OE*^ and *OsGRP3*^*KD*^ plants suggests that OsGRP3 is involved in the regulation of the ROS related genes. RNA-immunoprecipitation analysis revealed the associations of OsGRP3 with *PATHOGENESIS RELATED GENE 5* (*PR5*), *METALLOTHIONEIN 1d* (*MT1d*), *4,5-DOPA-DIOXYGENASE* (*DOPA*), and *LIPOXYGENASE* (*LOX*) transcripts. The half-life analysis showed that *PR5* transcripts decayed slower in *OsGRP3*^*OE*^ but faster in *OsGRP3*^*KD*^, while *MT1d* and *LOX* transcripts decayed faster in *OsGRP3*^*OE*^ but slower in *OsGRP3*^*KD*^ plants. H_2_O_2_ accumulation was reduced in *OsGRP3*^*OE*^ and increased in *OsGRP3*^*KD*^ plants compared to non-transgenic plants (NT) under drought stress.

**Conclusion:**

*OsGRP3* plays a positive regulator in rice drought tolerance and modulates the transcript level and mRNA stability of stress-responsive genes, including ROS-related genes. Moreover, *OsGRP3* contributes to the reduction of ROS accumulation during drought stress. Our results suggested that *OsGRP3* alleviates ROS accumulation by regulating ROS-related genes’ mRNA stability under drought stress, which confers drought tolerance.

**Supplementary Information:**

The online version contains supplementary material available at 10.1186/s12284-021-00473-0.

## Background

RNA–binding proteins play important roles in cellular processes by regulating gene expression at post-transcriptional levels, which involves mRNA stability, RNA splicing as well as RNA transport. Since the plant glycine-rich (GR) proteins were first discovered in petunia 30 years ago (Condit and Meagher 1986), multi-functional roles of these proteins have been reported in development and response to several biotic and abiotic stimuli (Ciuzan et al. 2015; Magdalena and Michal 2018). Glycine-rich proteins are categorized into four classes based on their protein structures. The biggest difference among the four classes is that class I to III have a signal peptide (SP) while class IV has an RNA-recognition motif (RRM) or cold shock domain (CSD) at the N-terminus. Class I represents only a simple structure containing SP and GR motifs, and class II is similar to class I except for the cysteine-rich region at the C-terminus. Genes belonging to class I and II function in plant cell wall development and organ growth (Park et al. 2001; Ringli et al. 2001). Class III has an additional oleosins domain between SP and GR regions. The glycine-rich RNA binding proteins (GRPs) are members of class IV subfamily possessing N-terminus RRMs in addition to C-terminal GR regions, and proposed to be involved in post-transcriptional regulation of its target transcripts (Kang et al. 2013; Ciuzan et al. 2015; Magdalena and Michal 2018).

Diverse biological activities of plant *GRP*s have been reported over the last decade (Kim et al. 2007b; Kim et al. 2008; Streitner et al. 2010; Yang et al. 2014). Plant *GRPs* are involved in seed germination, seedling growth, flowering time, and stress tolerance (Fusaro et al. 2007; Kim et al. 2008; Kim et al. 2010; Löhr et al. 2014; Yang et al. 2014). The Arabidopsis *AtGRP7*, one of the most-well characterized *GRP*s, regulates gibberellin (GA)-mediated stem growth by down-regulating GA biosynthesis (Löhr et al. 2014). Also, the heterologous expression of *AtGRP7* complemented the cold-sensitive phenotype of *E. coli* strain BX04, which lacks four cold shock proteins (Kim et al. 2007c). Similarly, overexpression of *AtGRP7* increases the tolerance of plants against low temperatures and freezing stress in Arabidopsis (Kim et al. 2008). *AtGRP7* also functions as a negative regulator for seed germination and seedling growth under dehydration and high salinity conditions (Kim et al. 2008).

To understand the molecular functions of GRPs, efforts to identify GRP-interacting RNAs were continued together with revealing the biological activity of GRPs. AtGRP7 possesses RNA chaperone activity and its N-terminal RRM and GR motifs are crucial for RNA chaperone activity (Kim et al. 2007c; Kwak et al. 2011). AtGRP7 preferentially interacts with U/C-rich RNA sequences (Meyer et al. 2017). Global transcript profiling also revealed that RNAs involved in biotic and abiotic stresses are accumulated in *AtGRP7* overexpressing plants (Streitner et al. 2010). Through RNA-immunoprecipitation (RNA-IP) assay, AtGRP7 appears to bind with transcripts of stress- and antioxidant-related genes in vivo, including *AtGRP7*, *COR15A/B*, *PR4*, *WRKY33,* and *MT2A* (Meyer et al. 2017). Besides, AtGRP7 and AtGRP8 can bind to each other’s mRNAs, affecting alternative splicing of each other (Schoning et al. 2008).

RNA-binding proteins can also shuttle mRNA from the nucleus to the cytoplasm (Kedersha et al. 2005; Thanin and Julia 2018). In Arabidopsis, AtGRP2 and 7 are localized in both nucleus and cytoplasm and involved in mRNA export (Fusaro et al. 2007; Kim et al. 2008; Lummer et al. 2011). There is also evidence showing that GRPs coordinately regulate the cytoplasmic processing of the mRNA targets under stress conditions. In Arabidopsis, AtGRP2, 7, and 8 were isolated as major components of messenger ribonucleoprotein complexes (mRNPs) under oxidative stress conditions (Schmidt et al. 2010). In mammal, the glycine-rich domain mediates the formation of cytoplasmic mRNP aggregates such as processing bodies and stress granules in which mRNA degradation and storage occur (Wolozin 2012).

Compared with progress on molecular functions of Arabidopsis GRPs, there are relatively few achievements on the functional characterization of rice GRPs. Early studies were focused on the tissue-specific expression patterns of *OsGRP*s (Xu et al. 1995; Liu et al. 2003). Kim et al. (2010) investigated the functional redundancy of Arabidopsis and rice *GRP*s in cold adaptation responses. They found that *OsGRP1, 4*, and *6* possess RNA chaperone activity and play a role in the cold adaptation process. Heterologous expression of rice *OsGRP4* and *OsGRP6* rescued a defect in the export of mRNA caused by a mutation in *AtGRP7* (Kim et al. 2010). These results suggest that GRPs in rice and Arabidopsis are functionally conserved and also function as RNA chaperones during the cold adaptation process. Different from their functional conservation in cold adaptation responses, GRP proteins play different functions in drought stress in Arabidopsis and rice. In Arabidopsis, *AtGRP7* is a negative regulator of seed germination and seedling growth under dehydration and high salinity conditions (Kim et al. 2008). They also found that stomatal closure was delayed in *AtGRP7* overexpressing plants under dehydration and high salinity conditions. Interestingly, overexpression of *AtGRP7* in rice conferred drought tolerance at both vegetative and reproductive stages (Yang et al. 2014). These results brought up the possibility that the roles of GRPs in drought response is different in Arabidopsis and rice.

In this study, we functionally characterized the rice glycine-rich protein *OsGRP3* and uncovered its roles in plants under drought stress. The expression of *OsGRP3* was found to be induced by ABA as well as drought stress. OsGRP3 proteins form cytoplasmic foci upon exposure to ABA and mannitol treatments. Through a combination of transcript profiling and RNA-IP-coupled qRT-PCR analysis, we screened *OsGRP3* target genes involved in ROS regulation and identified several OsGRP3-associating target mRNA transcripts. Furthermore, half-life analysis revealed that *OsGRP3* has roles in regulating the stability of its target mRNA, thus altering the tolerance of plants to drought stress. Taken together, we show that *OsGRP3* has important roles in drought tolerance by modulating the stability of its target transcripts involved in drought response and ROS regulation.

## Results

### OsGRP3 Is a Drought-Inducible Glycine-Rich-RNA Binding Protein

**Fig. 1:**
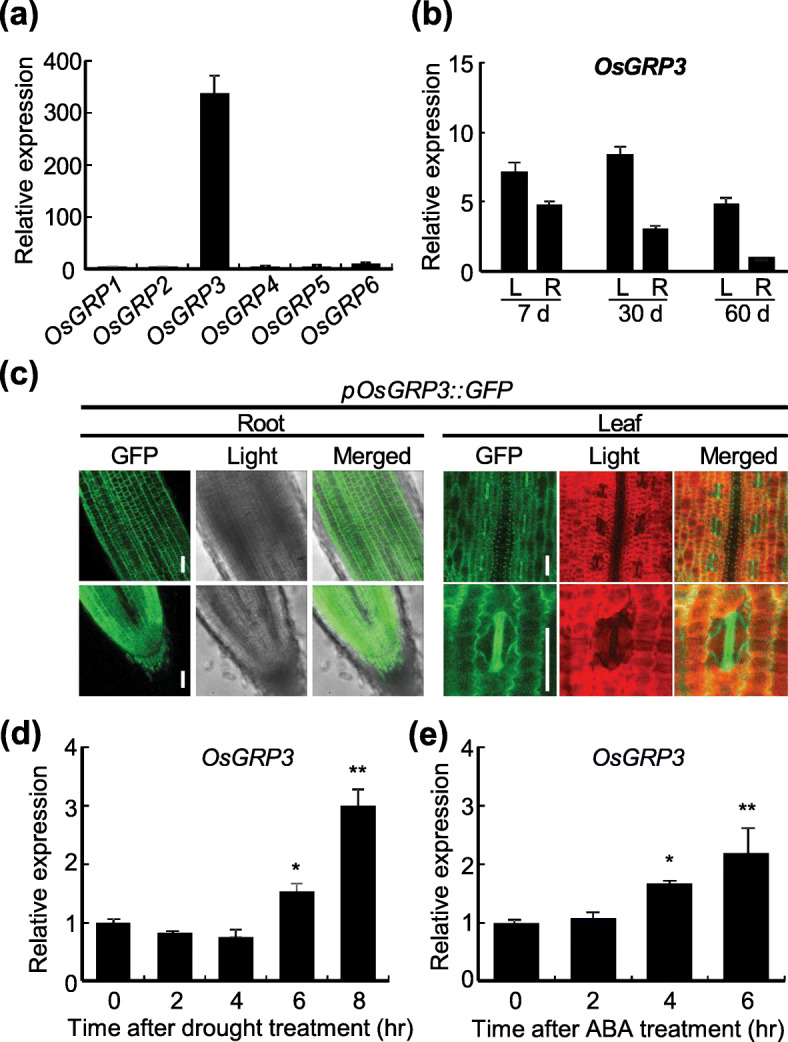
Expression of *OsGRP3* in rice plants under drought conditions. **a** qRT-PCR analysis was performed for measuring endogenous expression levels of six annotated *OsGRP*s using RNAs extracted from leaves of two-week-old rice seedlings. **b** Expression levels of *OsGRP3* in leaves and roots of rice plants at different developmental stages were analyzed using qRT-PCR. Rice *UBIQUITIN* (*OsUBI*) was used as an internal control for normalization. **a** and **b** Data represent the mean value ± standard deviation (SD) (*n* = 3, biological replicates). **c** The activity of *OsGRP3* promoter was analyzed by detecting GFP fluorescence in two-week-old *pOsGRP3::GFP* transgenic plants. Bar = 30 μm. Two-week-old rice seedlings were exposed to **d** drought stress and **e** 0.1 mM ABA. Leaves of rice seedlings were harvested at an indicated time points after the treatments. Rice *UBIQUITIN* (*OsUBI*) was used as an internal control for normalization. **d** and **e** Data represent the mean value ± standard deviation (SD) (*n* = 3, biological replicates). Asterisks indicate a statistically significant difference compared with 0 h. *P < 0.05, **P < 0.01; One-way ANOVA

To characterize the function of *OsGRP3* in rice, we first analyzed the amino acid sequences of rice GRPs and compared it against publicly available Arabidopsis GRP sequences. The phylogenetic analysis formed a clade consisting of *OsGRP3* and Arabidopsis *AtGRP1*, *AtGRP7* and *AtGRP8* (Fig. S1a). The RRM was highly conserved in both rice and Arabidopsis GRPs, but the length of the glycine-rich domain was diverse between the analyzed protein sequences (Fig. S1a). OsGRP3 possessed an RRM motif with a relatively longer glycine-rich domain (Fig. S1b). Among 6 annotated *OsGRP*s, *OsGRP3* expression was significantly higher (Fig. 1a). *GRP*s are reported to show tissue- and developmentally-specific expression patterns (Magdalena and Michal 2018). Thus, we examined the expression patterns of *OsGRP3* in various tissues at different developmental stages. *OsGRP3* expression was detected in all tested tissues (Fig. 1b and S2). To investigate spatial expression patterns of *OsGRP3* in detail, we generated transgenic rice plants expressing *GFP* under the control of the *OsGRP3* promoter (*pOsGRP3::GFP*) (Fig. 1c). GFP fluorescence was widely detected in both leaves and roots of rice plants with high GFP fluorescence in guard cells (Fig. 1c). These data indicate that *OsGRP3* is abundantly expressed in both leaves and roots of rice plants. We next analyzed expression patterns of *OsGRPs* in rice plants when exposed to drought conditions. *OsGRP3* exhibited a positive response against drought (air-drying) (Fig. 1d) as well as *OsGRP2* and *OsGRP4* (Fig. S3). ABA treatments also triggered a positive response in *OsGRP3* expression suggesting that its expression follows an ABA-dependent pathway (Fig. 1e). Taken together, these results indicate that *OsGRP3* is highly expressed in rice plants and is further induced by drought and ABA treatments.

### Overexpression of *OsGRP3* Confers Drought Tolerance in Rice

**Fig. 2:**
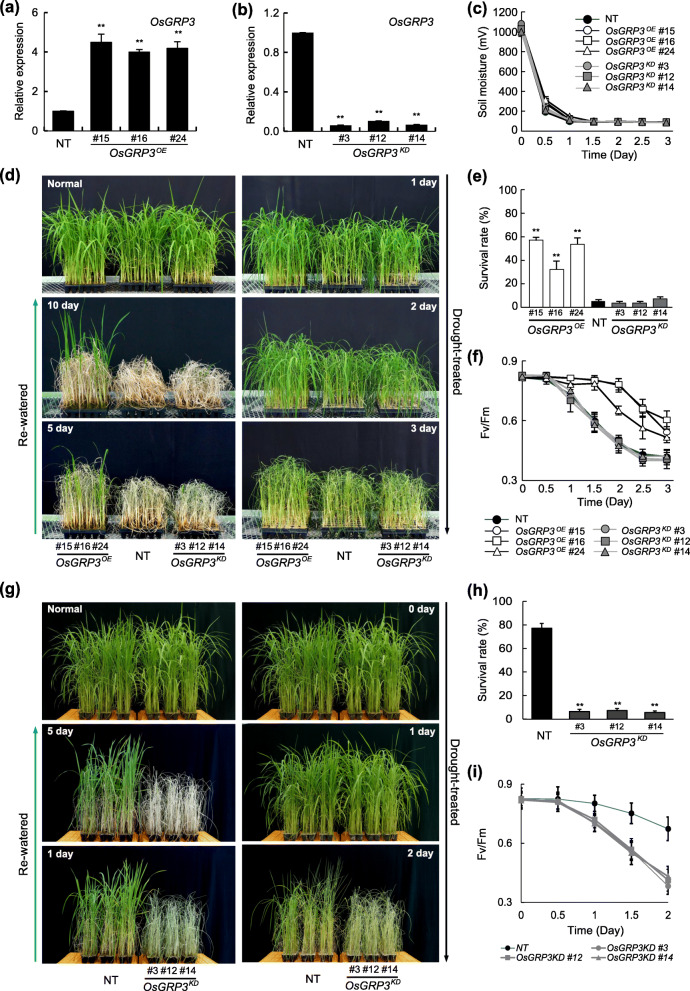
Overexpression of *OsGRP3* enhances drought tolerance in rice. Relative expression levels of *OsGRP3* in OsGRP3 overexpressing (*OsGRP3*^*OE*^) (**a**) and RNAi-mediated *OsGRP3* suppressing (*OsGRP3*^*KD*^) (**b**) transgenic plants. Total RNAs were extracted from leaves of two-week-old rice seedlings and used for qRT-PCR analysis. Rice *UBIQUITIN* (*OsUBI*) was used as an internal control for normalization. **a** and **b** Data represent the mean value ± standard deviation (SD) (*n* = 3, biological replicates). **c** Soil moisture content was monitored during drought treatment. Data represent the mean value ± standard deviation (SD) of 30 independent measurements performed at different locations of pots. **d** The drought-induced symptoms of non-transgenic (NT), *OsGRP3*^*OE*^ and *OsGRP3*^*KD*^ transgenic plants during drought stress and re-watering were visualized by taking pictures at indicated time points. **e** The survival rate of NT, *OsGRP3*^*OE*^ and *OsGRP3*^*KD*^ transgenic plants was calculated by counting the number of plants recovered from drought stress after re-watering. **f** Photochemical efficiency was analyzed by measuring the leaf chlorophyll fluorescence (F_v_/F_m_) of non-transgenic (NT), *OsGRP3*^*OE*^ and *OsGRP3*^*KD*^ transgenic plants during drought treatments. **g** The drought-induced symptoms of NT, *OsGRP3*^*OE*^ and *OsGRP3*^*KD*^ transgenic plants during drought stress and re-watering were visualized by taking pictures at indicated time points. **h** The survival rate of NT, *OsGRP3*^*OE*^ and *OsGRP3*^*KD*^ transgenic plants was calculated by counting the number of plants recovered from drought stress after re-watering. **i** F_v_/F_m_ value of NT and *OsGRP3*^*KD*^ transgenic plants during drought treatments. Value for each time point represents the mean ± standard deviation (SD) (*n* = 30, number of plants). Asterisks indicate a statistically significant difference compared with NT. **P < 0.01; One-way ANOVA

To investigate the physiological functions of *OsGRP3* in drought stress response, we generated transgenic plants overexpressing *OsGRP3* (*OsGRP3*^*OE*^) and RNAi-mediated knockdown lines (*OsGRP3*^*KD*^). To generate gene specific knockdown transgenic plants, we used *OsGRP3*-specific fragment designed at 3′ untranslated region of 173 bps. Thirty independent transgenic lines from each expression system were screened and shortlisted normally growing plants to eliminate the possible effects of somaclonal variations. Based on the expression levels of *OsGRP3,* three single-copy lines were selected from each expression system for further analysis. The *OsGRP3*^*OE*^ transgenic plants showed approximately 4-fold higher expression of *OsGRP3* than non-transgenic (NT) control plants (Fig. 2a) while *OsGRP3*^*KD*^ transgenic plants showed approximately 10-fold lower expression than in NT plants (Fig. 2). To evaluate the performance of the plants under drought conditions, selected transgenic plants (T3) and NT plants were grown in a greenhouse for 4 weeks and subjected to drought stress by withholding water for up to 3 days. Soil moisture content during drought treatments was monitored to make sure that stress treatments were uniformly applied (Fig. 2c). Drought-induced visual symptoms such as leaf rolling and wilting appeared earlier in NT than in *OsGRP3*^*OE*^ transgenic plants (Fig. 2d and S4) while *OsGRP3*^*KD*^ transgenic plants were indistinguishable from NT plants during drought treatments (Fig. 2d). After re-watering, *OsGRP3*^*OE*^ plants started to recuperate from drought-induced damages while NT and *OsGRP3*^*KD*^ plants continuously withered (Fig. 2d and S4). The enhanced tolerance of *OsGRP3*^*OE*^ transgenic plants to drought stress was further supported by a higher survival rate compared to NT plants (Fig. 2e). Since drought stress negatively affects photosynthetic efficiency of plants (Pinheiro and Chaves 2010), the degree of drought tolerance was further analyzed by measuring photochemical efficiency (*Fv/Fm*) of photosystem II in plants. *Fv/Fm* value in NT plants started to decrease 1 day after drought treatments and continued to decrease during the drought treatments (Fig. 2f). *OsGRP3*^*OE*^ plants, on the other hand, showed a slower decrease of *Fv/Fm* value than NT control plants during the drought treatments (Fig. 2f). Next, we applied the mild-drought condition by withholding water for up to 2 days to compare to drought phenotype between *OsGRP3*^*KD*^ plants and NT plants. Drought-induced visual symptoms appeared earlier in *OsGRP3*^*KD*^ plants than NT plants (Fig. 2g). The reduced tolerance of *OsGRP3*^*KD*^ transgenic plants to drought stress was further supported by a significant lower survival rate compared to NT plants (Fig. 2h), and *OsGRP3*^*KD*^ plants, on the other hand, showed a faster decrease of *Fv/Fm* value than NT control plants during the drought treatments (Fig. 2i). Taken together, these results indicate that overexpression of *OsGRP3* improves and knock-down of *OsGRP3* reduce drought tolerance in plants.

### OsGRP3 Proteins Are Localized in RNA Processing Sites

**Fig. 3:**
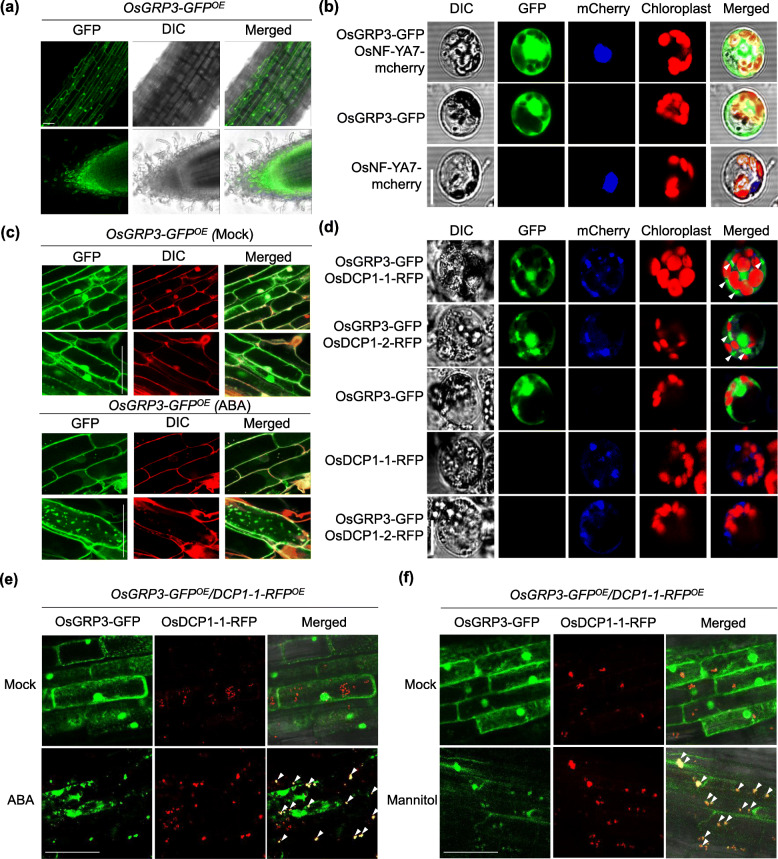
Change of subcellular localization of OsGRP3 in response to abiotic stresses. **a** Subcellular localization of OsGRP3 was visualized by detecting GFP fluorescence in roots of *OsCc1::OsGRP3-GFP* (*OsGRP3-GFP*^*OE*^) transgenic plants under normal growth conditions. Bar = 30 μm. **b** OsGRP3-GFP and OsNF-YA7-mCherry were transformed into rice protoplasts. Fluorescence of GFP, mCherry, and chloroplast was analyzed using confocal microscopy. Bar = 10 μm. **c** GFP Fluorescence in roots of *OsGRP3-GFP*^*OE*^ transgenic plants was analyzed 6 h after treatment with ABA. Propidium iodide (PI) staining was used for visualizing outlines of root cells. Bar = 30 μm **d** Fluorescence of GFP, RFP and chloroplast were analyzed in the transformed protoplasts 6 h after ABA treatment using confocal microscopy. Bar = 10 μm. GFP Fluorescence in roots of *OsGRP3-GFP*^*OE*^*/ DCP1–1-RFP*^*OE*^ transgenic plants was analyzed 6 h after treatment with ABA (**e**) and Mannitol (**f**). Bar = 30 μm. White arrows indicate co-localized sites of OsGRP3 and OsDCP1

Functions of RNA-binding proteins differ depending on their subcellular localization (Thanin and Julia 2018). To determine the subcellular localization of OsGRP3, we generated transgenic plants expressing OsGRP3-GFP fusion protein under the control of the *OsCc1* constitutive overexpression promoter (*OsGRP3-GFP*^*OE*^). Drought tolerance of *OsGRP3-GFP*^*OE*^ transgenic plants was confirmed so that GFP tagging did not disturb the function of OsGRP3 (Fig. S5). GFP fluorescence signals were detected in roots (Fig. 3a) and leaf nuclei (Fig. S5d) of the transgenic plants. We also observed GFP signals in the cytoplasmic membrane of both leaf and root tissues that is typical patterns shown by cytosolic proteins (Fig. 3a and S5d). To confirm the nuclear localization of OsGRP3, we expressed OsGRP3-GFP fusion protein under control of cauliflower mosaic virus *35S* promoter (*35S::OsGRP-GFP*) in rice protoplasts together with OsNF-YA7-mCherry fusion protein as a positive control for nuclear localization (Lee et al. 2015). When OsGRP3-GFP was co-expressed with OsNF-YA7-mCherry in rice protoplasts, fluorescent signals of OsGRP3-GFP overlapped with OsNF-YA5-mCherry but it was also detected in cytosol confirming that OsGRP3 is localized in both nucleus and cytosolic region of plant cells (Fig. 3b).

Since environmental stresses affect not only the expression of RNA-binding proteins but also their localizations (Bogamuwa and Jang 2013; Jan et al. 2013), we investigated how OsGRP3 behaves when plants are exposed to stress conditions. When treated with ABA, GFP fluorescence of *OsGRP3-GFP* ^*OE*^ plants, which were highly concentrated in the nucleus, appeared to disperse into the cytoplasm and formed cytoplasmic foci both in roots (Fig. 3c and S6a) and leaves (Fig. S6b). These foci started to form when ABA concentration was over 10 μM (Fig. S6a). Similarly, *OsGRP3-GFP* ^*OE*^ plants also showed distinct foci formation in the cytoplasm after mannitol treatments (Fig. 3f).

Since the localization of OsGRP3 in cytoplasmic foci was only detected under stress conditions, we hypothesized that OsGRP3 is transported into processing bodies (P-bodies) or stress granules under stress conditions. P-bodies and stress granules are dynamic cytosolic aggregates induced under stress conditions that play important roles in the regulation of mRNA stability (Thanin and Julia 2018). To determine whether OsGRP3 is associated with either P-bodies or stress granules, we constructed vectors expressing two rice orthologs of Arabidopsis DECAPPING 1 (DCP1) (OsDCP1–1 and OsDCP1–2) fused to RFP (OsDCP1–1-RFP and OsDCP1–2-RFP) and a rice ortholog of Arabidopsis POLY (A) BINDING PROTEIN 8 (OsPABP8) fused to RFP (OsPABP8-RFP) for visualizing P-bodies and stress granules, respectively. We then co-transformed OsGRP3-GFP into rice protoplasts together with OsDCP1-RFP or OsPABP8-RFP. GFP fluorescence of OsGRP3-GFP clearly overlapped with RFP fluorescence of OsDCP1-RFP after ABA (Fig. 3d) and mannitol treatments (Fig. S7). These co-localization of OsGRP3 in protoplasts was further verified by using transgenic roots co-overexpressing *OsGRP3-GFP* and *OsDCP1–1-RFP*. Consistent with results from protoplasts, OsGRP3 and OsDCP1–1 were co-localized at cytoplasmic foci (P-body) upon ABA and mannitol treatments (Fig. 3e and f). Unexpectedly, no clear cytoplasmic foci formation of OsPABP8-RFP was detected in rice protoplasts after both ABA and mannitol treatments, while RFP fluorescence of OsPABP8-RFP was detected in cytoplasmic foci when the protoplasts were treated with heat stress (Fig. S8). Under heat stress, OsGRP3-GFP and OsPABP8-RFP showed co-localization in the cytosolic foci (Fig. S8). Collectively, these results suggest that OsGRP3 associated with P-bodies as well as stress granules in response to abiotic stresses.

### Identification of Genes Regulated by *OsGRP3*

**Fig. 4:**
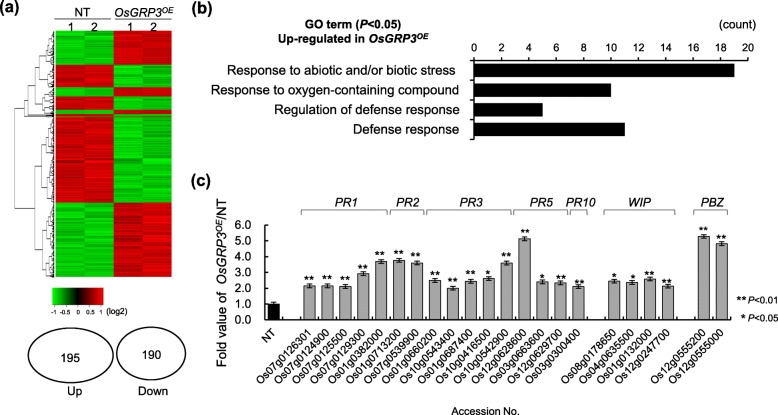
Identification of differentially expressed genes by OsGRP3 overexpression. RNA sequencing analysis were performed using RNAs extracted from two-week-old non-transgenic (NT) and OsGRP3 overexpressing (*OsGRP3*^*OE*^) transgenic plants. **a** Heat map analysis of differentially expressed genes (DEG) between NT and *OsGRP3*^*OE*^ transgenic plants. Van diagram indicates the number of up- and down-regulated DEGs in OsGRP3^OE^ plants than NT plants. **b** Gene ontology (GO) analysis of the up-regulated genes in *OsGRP3*^*OE*^ transgenic plants. **c** Fold increase the value of the up-regulated genes classified as biotic stress and defense categories through GO analysis. Data represent the mean value ± standard deviation (SD) (*n* = 2, biological replicates). Asterisks indicate a statistically significant difference compared with NT. *P < 0.05, **P < 0.01; Student t-test

To identify the genes in the *OsGRP3*-mediated drought tolerance pathway, we performed RNA-sequencing analysis to isolate gene expression profiles in *OsGRP3*^*OX*^ and *OsGRP3*^*KD*^ plants compared with NT plants. A cutoff change of at least two-fold was used to isolate up- and down-regulated genes by *OsGRP3*. The analysis revealed that *OsGRP3*^*OX*^ plants contained 195 up-regulated and 190 down-regulated genes compared with NT plants (Fig. 4a and Table S2). Among the 195 up-regulated transcripts, 45 genes belonged to the gene ontology (GO) category of response to abiotic and/or biotic stress and defense (Fig. 4b). The majority of these genes were categorized into defense- and stress-related genes and interestingly many *pathogenesis-related* (*PR*) genes were up-regulated (Fig. 4c and Table S3). This high frequency of defense- and stress-related genes affected by *OsGRP3* overexpression suggests that *OsGRP3* is closely associated with biotic and abiotic stress responses.

We then isolated genes showing opposite expression patterns between *OsGRP3*^*OX*^ and *OsGRP3*^*KD*^ plants as candidate genes directly regulated by *OsGRP3* (Table S2 and S4). Through these criteria, we finally isolated 15 genes as target candidates in the *OsGRP3*-mediated drought tolerance pathway (Table 1). Interestingly, six genes showing a positive correlation with the expression level of *OsGRP3* were associated with plant stress responses, including four *PR* genes, *LATE EMBRYOGENESIS ABUNDANT PROTEIN 17* (*LEA17*), and *EXOCYST SUBUNIT EXO70 FAMILY PROTEIN FX1* (*FX1*)*.* On the other hand, eight genes showing negative correlation with *OsGRP3* expression level included *PEROXIDASE 1* (*POX1*)*, METALLOTHIONEIN 1d* (*MT1d*)*, CAROTENOID 9, 10-CLEAVAGE DIOXYGENASE 1* (*CCD1*)*, 4,5-DOPA DIOXYGENASE EXTRADIOL-LIKE PROTEIN* (*DOPA*)*, LEUCINE-RICH REPEAT DOMAIN CONTAINING PROTEIN* (*LRR*)*, LIPOXYGENASE* (*LOX*) and S-ADENOSYLMETHOININE SYNTHETASE 1 (*SAM1*) that were associated with reactive oxygen species (ROS) regulations (Nisar et al. 2015; You and Chan 2015; Liang et al. 2019). These results indicate that *OsGRP3* affects the gene expression involved in ROS regulation.

**Table 1:** List of candidate genes regulated by *OsGRP3*

Gene_ID	Gene	Abbreviation	Fold value *OsGRP3*^*OE*^ /NT^a^	*P* value *OsGRP3*^*OE*^ /NT^b^	Fold value *Osgrp3*^*KD*^ /NT^a^	*P* value *Osgrp3*^*KD*^ /NT^b^
**< Genes positively regulated by** ***OsGRP3*>**						
Os07g0559000	Non-protein coding transcript	*NP*	7.3	0.00	−2.5	0.05
Os03g0670700	OsGRP3	*OsGRP3*	3.1	0.13	−82.9	0.00
Os05g0369900	Exocyst subunit EXO70 family protein	*FX1*	2.6	0.01	−2.7	0.01
Os03g0322900	Late embryogenesis abundant protein 17	*LEA17*	2.4	0.07	−2.1	0.03
Os12g0629700	PR5	*PR5*	2.3	0.01	−3.4	0.02
Os07g0124900	OsPR1–071	*PR1*	2.2	0.07	−4.7	0.00
Os07g0126301	A paralog of OsPR1–071	*PR1*	2.2	0.07	−4.7	0.00
Os07g0125500	A paralog of OsPR1–071	*PR1*	2.1	0.07	−4.6	0.00
**< Genes negatively regulated by** ***OsGRP3*>**						
Os01g0263300	Peroxidase 1	*POX1*	−3.5	0.01	3.7	0.02
Os12g0571100	Metallothionein 1d	*MT1d*	−3.0	0.02	2.4	0.04
Os08g0371200	Carotenoid 9,10-cleavage dioxygenase 1	*CCD1*	−2.4	0.00	2.1	0.04
Os01g0878900	4,5-DOPA dioxygenase extradiol-like protein	*DOPA*	−2.4	0.02	2.4	0.03
Os01g0162300	Leucine-rich repeat protein	*LRR*	−2.3	0.02	2.3	0.01
Os10g0361000	Lipoxygenase	*LOX*	−2.2	0.06	5.0	0.00
Os10g0580900	Conserved hypothetical protein		−2.0	0.03	2.1	0.12
Os01g0293000	S-adenosylmethionine synthetase 1	*SAM1*	−2.0	0.03	9.0	0.00

^a^The mean of data obtained from two biological replicates. ^b^*P* value based on one-way ANOVA

### OsGRP3 Interacts with its Target RNAs and Regulates their Stability

**Fig. 5:**
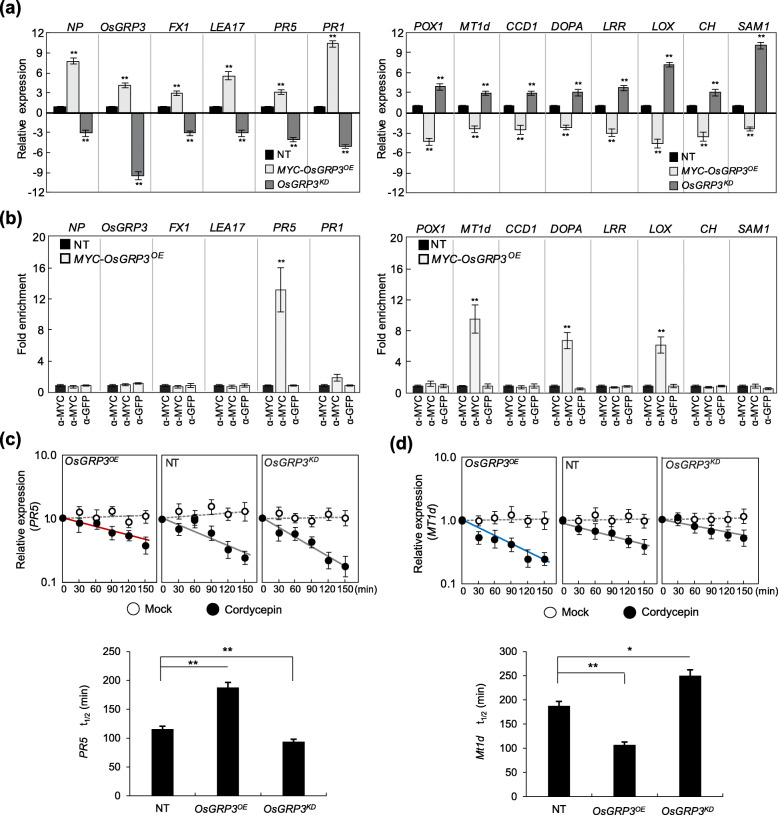
OsGRP3 affects the stability of its binding transcripts **a** Validation of expression levels of DEGs isolated from RNA sequencing analysis in non-transgenic plants (NT), *MYC*-*OsGRP3* overexpressing (*MYC-OsGRP3*^*OE*^) and RNAi-mediated *OsGRP3* suppressing (*OsGRP3*^*KD*^) transgenic plants by qPCR analysis. Total RNAs were isolated from leaves of two-week-old NT, *MYC-OsGRP3*^*OE*^ and *OsGRP3*^*KD*^ transgenic plants. Rice *UBIQUITIN* (*OsUBI*) was used as an internal control for normalization. **b** Analysis of OsGRP3 interaction with the transcripts of the selected DEGs. Leaves of two-week-old *MYC-OsGRP3*^*OE*^ and NT plants were used for RNA-IP experiments. The anti-GFP antibody was used as a negative control for the anti-MYC antibody. Fold enrichment was normalized to the value of NT plants. **a** and **b** Data represent the mean value ± standard deviation (SD) (*n* = 3, biological replicates). Half-life measurement of **c** *PR5* and **d** *Mt1d* transcripts. Two-week-old NT, *OsGRP3*^*OE*^ and *OsGRP3*^*KD*^ transgenic plants were pre-treated with 1 mM cordycepin for 30 min. Plants were then harvested every 30 min after mock or cordycepin treatments. Total RNAs extracted from the harvested samples were applied for qRT-PCR analysis. **c** and **d** Data represent the mean value ± standard deviation (SD) of three biological replicates. Half-lives of mRNA were calculated based on equation as shown in Table S5. Asterisks indicate a statistically significant difference compared with NT. **P < 0.01; One-way ANOVA

Since GRP3 contained two conserved RNA-recognition motifs, which are crucial for interaction with RNAs (Streitner et al. 2012), we hypothesize that OsGRP3 forms an in vivo complex with its target mRNAs. To prove the hypothesis, we performed RNA-IP using transgenic rice plants expressing *OsGRP3* translationally fused with MYC epitope under control of the *GOS2* promoter (*GOS2::MYC-OsGRP3; MYC-OsGRP3*^*OE*^) to isolate in vivo OsGRP3-RNA complex. Expression patterns of the selected DEGs (Table 1) in *MYC-OsGRP3*^*OE*^ plants and drought tolerance of transgenic plants were similar to that of *OsGRP3*^*OE*^ plants (Fig. 5a and S5a) confirming that MYC epitope tagging did not disturb the function of OsGRP3. OsGRP3-RNA in vivo complex was then isolated by RNA-IP using MYC antibody. qRT-PCR analysis was performed to examine the enrichment of OsGRP3 on selected transcripts. Two of the 3 duplicate transcripts of *PR1* genes were excluded since *PR1* genes showed duplication in 3 separate loci in chromosome 7 producing three identical transcripts. Among the 6 genes positively regulated by *OsGRP3*, levels of *PR5* transcripts were the highest in OsGRP3-RNA complex (Fig. 5b). On the other hand, transcripts of three genes negatively regulated by *OsGRP3* were concentrated by immunoprecipitation of OsGRP3 (Fig. 5b). These were *MT1d*, *DOPA* and *LOX* transcripts with enrichment values of 12-, 7- and 5-fold higher than control, respectively. These results confirmed direct or indirect association of the OsGRP3 protein with target transcripts.

To further investigate the function of *OsGRP3* in the regulation of its binding mRNAs, the half-lives of OsGRP3 bound transcripts (*PR5*, *MT1d, DOPA,* and *LOX)* were determined in *OsGRP3*^*OE*^_,_ *OsGRP3*^*KD*^ and NT plants (Fig. 5c, S9, and Table S5). The transcriptional inhibitor Cordycepin (3′ deoxyadenosine) was used to exclude the change of transcripts level caused by de novo transcription. There was no significant change in levels of all tested transcripts without cordycepin treatments, but the levels of the transcripts started to decrease after cordycepin treatments (Fig. 5c and S9). The half-life of *PR5* transcripts in NT plants was 115 min and increased to 187 min in *OsGRP3*^*OE*^ but decreased to 93 min in *Osgrp3*^*KD*^ plants (Fig. 5c). In contrast, the half-life of *MT1d* was only 107 min in *OsGRP3*^*OE*^ plants, while those were 187 and 250 min in NT and *OsGRP3*^*KD*^ plants, respectively (Fig. 5d). Similarly, *LOX* transcripts were less stable in *OsGRP3*^*OE*^ yet more stable in *OsGRP3*^*KD*^ compared with NT plants (Fig. S9b and Table S5). *DOPA* transcripts, however, were more stable in *OsGRP3*^*OE*^ plants despite its lower expression level in *OsGRP3*^*OE*^ plants (Fig. S9a and Table S5). Collectively, these results show that OsGRP3 regulates its target mRNAs at the post-transcriptional level by affecting their stabilities.

### Effect of Drought Stress on the Functions of OsGRP3

**Fig. 6:**
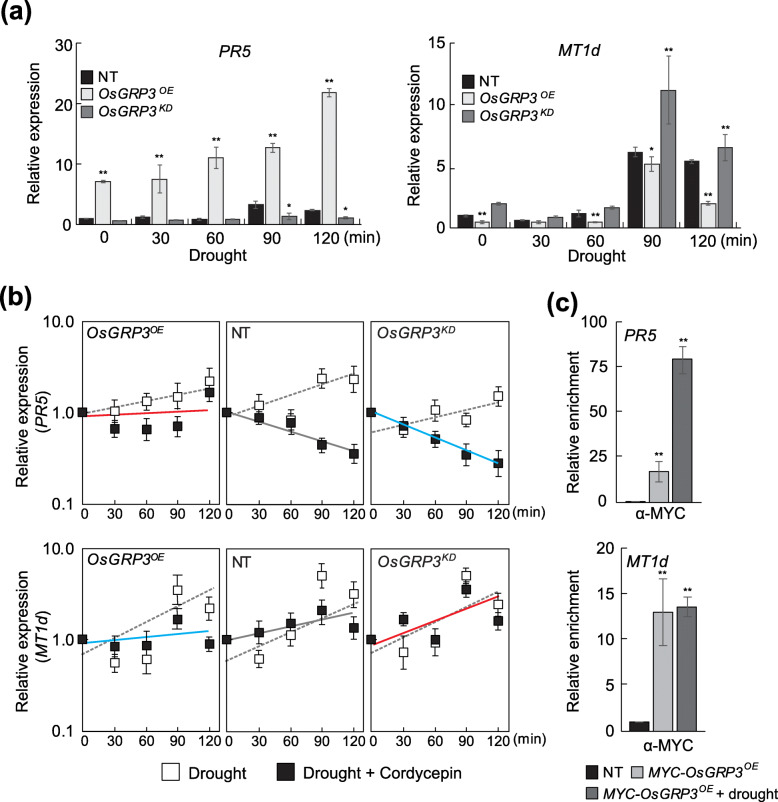
Effects of drought on the function of OsGRP3. **a** Expression patterns of *PR5* and *MT1d* were analyzed under drought conditions. Two-week-old non-transgenic plants (NT), *OsGRP3* overexpressing (*OsGRP3*^*OE*^) and RNAi-mediated *OsGRP3* suppressing (*OsGRP3*^*KD*^) transgenic plants were air-dried and harvested at indicated time points. Total RNAs isolated from harvested leaves were analyzed by qRT-PCR. Rice *UBIQUITIN* (*OsUBI*) was used as the internal control for normalization. **b** The stability of *PR5* and *Mt1d* transcripts was analyzed under drought conditions. Two-week-old NT, *OsGRP3*^*OE*^ and *OsGRP3*^*KD*^ transgenic plants were pre-treated with distilled water or 1 mM cordycepin for 30 min. Plants were then treated with drought stress and harvested every 30 min after the treatments. Total RNAs extracted from the harvested samples were applied for qRT-PCR analysis. **c** Analysis of OsGRP3 interaction with *PR5* and *MT1d* transcripts under drought conditions. For analyzing the interaction of OsGRP3 with *PR5* and *Mt1d* transcripts, two-week-old *MYC-OsGRP3*^*OE*^ plants were harvested 2 h after drought treatments and applied for RNA-IP experiments. NT plants were used as a negative control. Fold enrichment was normalized to the value of NT plants. **a**-**c** Data represent the mean value ± standard deviation (SD) (*n* = 3, biological replicates). **a** and **c** Asterisks indicate a statistically significant difference compared with NT. *P < 0.05, **P < 0.01; One-way ANOVA

Since both the expression of *OsGRP3* and its subcellular localization was altered under stress conditions (Figs. 1d and 3), we examined the effects of drought stress on the functions of *OsGRP3* by monitoring the stability of its target mRNAs under drought conditions (air-drying). qRT-PCR analysis revealed that drought treatments induced expression of *PR5* and *MT1d* but not *DOPA* and *LOX* genes in rice plants (Fig. 6a and S10). Thus, we focused on the regulation of *PR5* and *MT1d* expression for explaining the functions of OsGRP3 under drought conditions. In *OsGRP3*^*OE*^ plants, *PR5* expression was higher than NT plants under normal conditions and further induced by drought treatments, while the drought-induced expression of *PR5* was significantly reduced in *OsGRP3*^*KD*^ plants. In contrast, drought inducible *MT1d* expression was lower in *OsGRP3*^*OE*^ plants but higher in *OsGRP3*^*KD*^ plants compared to NT plants (Fig. 6a). We then examined expression patterns of *PR5* and *MT1d* in plants treated with both drought and cordycepin to determine the changes in the stability of these genes under drought conditions (Fig. 6b). The cordycepin treatments significantly reduced *PR5* transcript levels in NT, indicating that transcription of the *PR5* gene is successfully inhibited by the treatments. Similarly, *PR5* transcripts were gradually reduced in *OsGRP3*^*KD*^ plants. Different from NT and *OsGRP3*^*KD*^ plants, *PR5* transcripts were initially reduced but increased later in *OsGRP3*^*OE*^ plants (Fig. 6b and Table S5). In contrast, the *MT1d* transcripts were more stable in *OsGRP3*^*KD*^ plants but less stable in *OsGRP3*^*OE*^ plants than NT plants under drought conditions (Fig. 6b and Table S5). We then examined the effect of drought treatments on the association of OsGRP3 with its target mRNAs. Drought treatments increased the transcript amount of *PR5* but not to *MT1d* retrieved by RIP assay (Fig. 6c). Taken together, *OsGRP3* differently regulates the stability of target mRNAs, and its regulation is affected by drought stress in rice plants.

### ROS Accumulation Levels Are Altered in OsGRP3 Transgenic Plants under Drought Stress Conditions

**Fig. 7:**
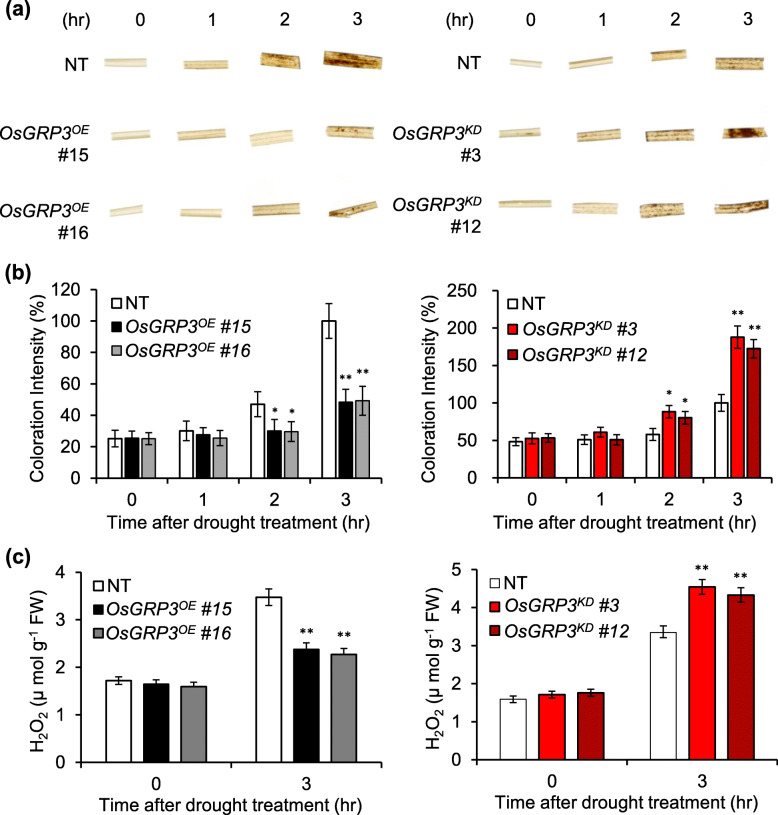
Assessment of relative H_2_O_2_ levels in *OsGRP3* transgenic rice plants under drought conditions. **a** Levels of H_2_O_2_ in leaves of two-week-old rice seedlings were visualized by DAB staining with time-course after drought treatment (airdrying). **b** Histograms depicting relative quantity of H_2_O_2_ in terms of their coloration intensity **c** Measurement of H_2_O_2_ levels in leaves of two-week-old rice seedlings after drought treatment (airdrying). The data represent the mean value ± standard deviation (SD) of three biological replicates (*n* = 3). Asterisks indicate a statistically significant difference compared with NT. *P < 0.05, **P < 0.01; One-way ANOVA

Majority of target genes of *OsGRP3* including *LEA17*, *POX1*, *MT1d*, *CCD1*, *DOPA*, and *LOX* are involved in regulation of ROS accumulation. Therefore, we checked the H_2_O_2_ accumulation levels in NT, *OsGRP3*^*OE*^, and *OsGRP3*^*KD*^ plants under drought conditions. To examine H_2_O_2_ levels, leaves of the NT and transgenic lines subjected to air-drying were stained with DAB (Fig. 7a) and quantified by densitometric scanning of the DAB stained leaves (Fig. 7b). Histochemical staining showed that under normal conditions, transgenic lines had similar H_2_O_2_ levels to NT. Notable increase in ROS levels in NT was observed 2 h after air-drying (Fig. 7a). However, the leaves of *OsGRP3*^*OE*^ showed significantly less accumulation of H_2_O_2_ (Fig. 7a and b; left panels) after air-drying. On the contrary, the leaves of *OsGRP3*^*KD*^ showed more ROS accumulation than that of NT (Fig. 7a and b; right panels). When we measured the H_2_O_2_ concentration of the plants, H_2_O_2_ levels of *OsGRP3*^*OE*^ and *OsGRP3*^*KD*^ plants were significantly lower and higher than those of NT plants, respectively, after air-drying. These results are consistent with the observed drought tolerance of *OsGRP3*^*OE*^ plants (Fig. 2). Taken together, OsGRP3 is involved in the regulation of H_2_O_2_ levels under drought stress conditions.

## Discussion

Characterization of glycine-rich RNA-binding proteins (GRPs) has revealed their important roles in development and response to environmental stresses (Lorkovic 2009; Magdalena and Michal 2018), but detailed molecular mechanisms governed by GRPs are still largely unknown. Potential involvement of GRPs in drought stress responses has been proposed in several studies, however, molecular mechanism of drought phenotype of GRPs still has been unexplored (Kim et al. 2008; Chen et al. 2010; Yao et al. 2016; Wang et al. 2018). Here we combined physiological and molecular approaches in elucidating the function of OsGRP3 under normal and stressed conditions. Overexpression of *OsGRP3* significantly enhanced and knock-down of *OsGRP3* reduced the drought tolerance of the transgenic plants (Fig. 2 and S4) suggesting that *OsGRP3* function as a positive regulator in drought tolerance. Arabidopsis *AtGRP7*, which belongs to the same clade with *OsGRP3* (Fig. S1), acts as a negative regulator of seed germination under dehydration conditions (Kim et al. 2008) and its heterologous expression in rice improves drought tolerance of the plants (Yang et al. 2014). Together with our findings, these results reinforced the idea that different GRP proteins regulate drought responses of plants in an opposite way.

Downstream genes up-regulated by *OsGRP3* were categorized into two major groups: defense response and stress-related genes (Table 1 and Fig. 4). The defense response genes up-regulated by *OsGRP3* overexpression included three *WRKY* transcription factors (*OsWRKY45,79* and *62*) and 19 *PR* genes (*OsPR1*, *2*, *5*, and *10*) (Table S3). Similar to our data, expression of *PR1*, *PR2,* and *PR5* expression was also up-regulated in Arabidopsis plants by overexpression of *AtGRP7* (Streitner et al. 2010). Besides, heterogeneous overexpression of *AtGRP2* and *AtGRP7* also induced the expression of *OsPR10a* in rice plants and showed drought tolerance (Yang et al. 2014)*.* These indicate that *GRP*-mediated regulation of *PR* genes is highly conserved in both Arabidopsis and rice plants.

So far, it has been known that *WRKY* and *PR* genes are main components of salicylic acid (SA) mediated plant defense mechanisms and also involved in plant abiotic stress mechanisms in many plants (Qiu and Yu 2009; Seo et al. 2011; Ali et al. 2018). *OsWRKY45* and *OsWRKY62* were identified as molecular interfaces of biotic and abiotic stress interaction (Seo et al. 2011) and overexpression of *OsWRKY45* enhanced tolerance to both drought and biotic stresses (Qiu and Yu 2009). *PR* genes also conferred tolerance to drought when overexpressed in plants, as reported in several studies (Liu et al. 2013; Wu et al. 2016; Li et al. 2019). Critically, overexpression of Arabidopsis *PR* genes (*PR1*, *PR2,* and *PR5*) and rice *OsPR10* improved the performance of plants under drought conditions (Wu et al. 2005; Liu et al. 2013). Moreover, mutation of *NPR1*, the master regulator of *WRKY* and *PR* gene expression, reduced drought tolerance in tomato (Li et al. 2019). These reports indicate that the observed drought tolerance in *OsGRP3*^*OE*^ plants could be mainly achieved due to the up-regulation of *WRKY* and *PR* genes.

The stress-inducible genes up-regulated by *OsGRP3* overexpression include *OsLEA17* and rice *PLASMA MEMBRANE PROTEIN 1* (*OsPM1*). LEA proteins are hydrophilic proteins involved in drought tolerance mechanisms of rice by increasing levels of osmolyte and soluble sugar and cell membrane stability, and by reducing ROS (Duan and Cai 2012; Kaur et al. 2017; Liang et al. 2019). *OsPM1* mediates ABA influx through the plasma membrane for ABA-dependent guard cell closure in plants (Yao et al. 2018). Thus, the up-regulation of the stress-inducible genes by *OsGRP3* overexpression may also constitute another mechanism to enhance drought tolerance.

Identifying the associated RNAs is crucial in understanding the molecular functions of RNA-binding proteins (RBPs). Higher enrichment of *PR5* mRNA in RNA-IP analysis confirmed the physical association of OsGRP3 with *PR5* mRNA (Fig. 5b). In addition, the stability of *PR5* transcripts increased in *OsGRP3*^*OE*^ but decreased in *OsGRP3*^*KD*^ plants (Fig. 5c and Table S5) and under drought conditions than under normal conditions (Figs. 5c, 6b, and Table S5). Moreover, a significant increase in the retrieved transcript quantity of *PR5* by OsGRP3 was detected under drought conditions (Fig. 6c). These results suggested that O

sGRP3 contributes to drought-induced stabilization of *PR5* mRNAs. Similar to our findings, *AtGRP7* positively regulates the expression of several *PR* genes in Arabidopsis, and RNA binding activity of *AtGRP7* is required for the up-regulation of *PR* genes (Streitner et al. 2010; Hackmann et al. 2014). Besides, it was found that AtGRP7 physically associates with *PR4* transcripts in Arabidopsis (Meyer et al. 2017). RBPs have been shown to function as regulators in post-transcriptional regulation of RNA metabolism, and some RBPs have RNA chaperone activity that prevents RNA misfolding or resolves misfolded RNAs (Kang et al. 2013). It has been reported that *OsGRP3* exhibits RNA chaperone activity during cold adaptation process (Kim et al. 2010). Interestingly, the three up-regulated paralogs of *PR1* in *OsGRP3*^*OE*^ plants seemed to be indirect targets of *OsGRP3* overexpression since no clear enrichment in RNA-IP was observed (Fig. 4b). However, it was reported that AtGRP7 also up-regulated *PR1* transcript without direct binding (Meyer et al. 2017). Therefore, *OsGRP*3, directly and indirectly, regulate the stability of target mRNAs of *PR* genes to confer drought tolerance in rice.

Transcriptome analysis of *OsGRP3*^*OE*^ and *OsGRP3*^*KD*^ transgenic plants revealed that the DEGs negatively regulated by *OsGRP3* are related to the regulation of antioxidant components such as metallothioneins and carotenoids in plants (Table 1). *METALLOTHIONEIN* genes encode low molecular antioxidants that regulate ROS under stress conditions (Zhu et al. 2009; Grennan 2011; Kumar et al. 2012; Han et al. 2014). *CCD1*, *POX1*, and *LOX* are involved in the regulation of cellular carotenoid content. CCD leads to the enzymatic turnover of C40 carotenoids into apocarotenoids which is a critical step in regulating carotenoid accumulation (Auldridge et al. 2006; Nisar et al. 2015). In addition, oxidation of carotenoid by POX1 and LOX is also required for maintaining carotenoid homeostasis in photosynthetic tissues (Auldridge et al. 2006; Walter and Strack 2011). Therefore, opposite expression pattern of these genes in *OsGRP3*^*OE*^ and *OsGRP3*^KD^ plants suggested that *OsGRP3* directly or indirectly involved in regulating cellular ROS level response to drought stress and H_2_O_2_ accumulation results support this idea (Fig. 7). Various abiotic stresses increase cellular ROS levels of plants leading to oxidative stress (Gill and Tuteja 2010; You and Chan 2015). Previous reports showed that Arabidopsis *atRZ-1a*, a GRP containing zinc finger motif, positively regulates ROS accumulation under salt and dehydration stresses (Kim et al. 2007a). These results suggested that GRPs were closely involved in the regulation of ROS levels under stress conditions.

Among the genes negatively regulated by OsGRP3, *OsMT1d*, *DOPA*, and *LOX* mRNA showed physical interactions with OsGRP3 in RNA-IP analysis (Fig. 4b). *MT* and *LOX* genes have also been found as interactors of AtGRP7 in RNA-IP analysis (Meyer et al. 2017). Our RNA half-life analysis revealed that *OsMT1d* and *LOX* transcripts were more stable in *OsGRP3*^*KD*^ transgenic plants and less stable in *OsGRP3*^*OE*^ transgenic plants than NT control plants (Figs. 5c, 6b, and S10). It has not been characterized by how GRPs negatively regulates the stability of target mRNAs. RNA binding proteins involved in mRNA turnover are transported into P-bodies and stress granules under stress conditions in plants (Pomeranz et al. 2010; Jan et al. 2013). It was thought that the movement of RNA binding proteins mediate the transportation of mRNA into P-bodies or stress granules for post-transcriptional controls (Thanin and Julia 2018). OsGRP3 proteins were detected in both the nucleus and cytoplasm under unstressed conditions and found in P-bodies after ABA and mannitol treatments (Fig. 3, S6 and S7). These observations suggest that the negative regulation of *MT1d* and *LOX* stability is related to the association of interacting partners with OsGRP3 in P-bodies. Interestingly, OsGRP3 regulated its target mRNAs, *PR5* and *MT1d*, oppositely. We could not find any proper reference in the literature for explaining such a dual mode of regulation. Our results showed that the amount of *PR5* mRNA bound by OsGRP was significantly increased while that of *MT1d* mRNA remained similar after drought treatments (Fig. 6c). It appears that OsGRP3 binding affinity towards target mRNAs is being changed by drought treatments. It is therefore possible that the different changes in OsGRP3 binding affinity to its interactors under drought stress conditions are responsible for the dual mode of regulation.

## Conclusion

In the present study, we provide physiological and molecular evidence indicating that the rice *OsGRP3* modulates the expression of stress- and ROS-related genes. *OsGRP3* transgenic plants showed that OsGRP3 negatively regulated ROS generation under drought condition compared to NT plants. We also identified that OsGRP3 physically interacts with its target mRNAs thereby regulating their stability. Taken altogether, OsGRP3 contributes to the enhanced tolerance of rice to drought stress by regulating mRNA stability of stress- and ROS related genes.

## Methods

### Plant Growth and Hormone Treatments

Rice seeds (*Oryza sativa* cv. Nipponbare) were sown on a Murashige–Skoog (MS) solid medium and incubated in the dark for 3 days at 28 °C. Then seedlings were transferred to a growth chamber with a light and dark cycle of 16 h light/8 h dark having a light intensity of 200 μmol m^− 2^ s^− 1^ and relative humidity of 70%. One-week-old seedlings were transferred to a liquid growth medium (Yoshida et al. 1976) and grown for 2 weeks for gene expression analysis. To examine ABA-dependent expression patterns of *OsGRP3*, seedlings were transferred to 50 ml tubes containing 100 μM ABA solution (Sigma, USA). For drought treatments, 3-week-old rice plants were air-dried at room temperature. Seedlings were harvested at indicated time points after ABA and drought treatment for RNA extraction.

### Plasmids Construction and Rice Transformation

To generate *OsGRP3* overexpressing plants, the coding sequence of *OsGRP3* (Os03g0670700) was amplified from rice (*Oryza sativa L. ssp. japonica* cv. Nipponbare) total RNAs using the Reverse Transcription System (Promega, USA) and PrimeSTAR HS DNA polymerase (Takara, Japan). The amplified *OsGRP3* coding sequence was cloned into rice transformation vector *p700* carrying *OsCc1* promoter for constitutive overexpression (*OsCc1::OsGRP3*) (Jang et al. 2002) and the final constructs were transformed into rice (*Oryza sativa* cv. Nakdong). For knock-down expression of *OsGRP3*, the inverted repeat gene cassette of *OsGRP3*-specific fragment was inserted into *p700* carrying *OsGOS2* promoter for constitutive overexpression (Jeong et al. 2010). The *OsGRP3*-specific fragment was designed at 3′ untranslated region of 173 bps in length and the final constructs were transformed into rice (*Oryza sativa* cv. Dongjin). To visualize the subcellular localization of OsGRP3 in rice plants, the coding sequence of OsGRP3 translationally fused with a green fluorescent protein (GFP) was inserted into *p700* vector carrying the *OsCc1* promoter (*OsCc1::OsGRP3-GFP*). To examine spatial expression patterns of *OsGRP3* in rice plants, the upstream 1886 bps sequence from the transcriptional start site of *OsGRP3* was amplified and used as an *OsGRP3* promoter. *OsGRP3* promoter was cloned into the *p700* vector containing the GFP coding sequence (*pOsGRP3::GFP*). The final constructs were transformed into rice (*Oryza sativa* cv. Dongjin) by Agrobacterium (LBA4404) mediated co-cultivation, as described previously (Jang et al. 1999). Primers used for plasmids construction were listed in Table S1. Copy numbers of the transgenic plants were determined by TaqMan Q-PCR (Thermo Fisher, USA) using probes specific for the *bar* gene. To analyze the copy number of the transgenic rice plants, genomic DNA was extracted from 2-week-old rice seedlings. The genomic DNA extracted from transgenic plants previously confirmed as a single inserted homozygous line was used as a control for the single-copy insertion. The selected single-copy insertion lines were self-fertilized, and homozygous transgenic lines were selected from T2 generations by examining segregation rates on MS media containing phosphinothricin (Duchefa, Netherlands). Three independent single-copy inserted homozygous plants were selected from over thirty individual lines and propagated in a rice paddy field at Kyungpook National University, Gunwi (128:34E/36:15 N), Korea.

### Confocal Microscopy

The GFP fluorescence of *OsCc1::OsGRP3-GFP* and *OsCc1::OsGRP3-GFP* / *OsGOS2::OsDCP1–1-RFP* transgenic rice plants was analyzed in leaves and 10 μM propidium iodide-stained roots of two-week-old transgenic plants using a Leica SP8 STED laser scanning confocal microscope (Leica, Germany). GFP and propidium iodide were excited at 488 nm, and then emission was detected between 512 and 560 nm for GFP and between 610 and 650 nm for propidium iodide, respectively. RFP was excited at 560 nm and then emission was detected between 580 and 620 nm.

### RNA Isolation and Quantitative Real-Time PCR Analysis

Rice tissues harvested at indicated time points were used for total RNA extraction using the Hybrid-R RNA purification kit (GeneAll, Korea) according to the manufacturer’s instructions. First-strand complementary DNA (cDNA) was synthesized from 2 μg of total RNAs using RevertAid M-MuLV Reverse Transcriptase (Thermo Scientific, USA). Real-time PCR analysis was carried out using 2X Real-Time PCR smart mix (SolGent, Korea) and EvaGreen (SolGent, Korea) in an Mx3000P Real-Time PCR system (Stratagene, USA). The PCR reactions were performed by initial denaturation at 95 °C for 15 min, followed by 40 cycles of 95 °C for 20 s, 60 °C for 20 s, and 72 °C for 30 s. Rice *Ubiquitin1* (Os06g0681400) was used as an internal control for normalization. Primers used for qRT-PCR analysis were listed in Table S1.

### Rice Protoplast Preparation and Transient Gene Expression

Rice seedlings (*Oryza sativa* cv. Dongjin) were grown in the dark for 10 days and transferred to light conditions for 10 h. Rice protoplast preparation and transient gene expression were performed as described previously (Shim et al. 2018). For transient expression of OsGRP3-GFP in rice protoplasts, OsGRP3-GFP fusion protein sequence was amplified from the *OsCc1::OsGRP3-GFP* construct and inserted into the *pHBT* vector carrying *35 s* promoter (Shim et al. 2018). Similarly*,* the coding sequence of *OsDCP1–1*, *OsDCP1–2* and *OsPABP8* omitting stop codon was inserted into a *pHBT* vector containing red fluorescent protein (RFP) for translation fusion of the inserts with RFP. Primers used for plasmid constructions were listed in Table S1. The constructs were then transformed into protoplasts using PEG-mediated transformation. The transformed protoplasts were treated with 0.1 mM ABA or 1.25 M mannitol and harvested 3 h after the treatments by centrifugation at 300 g for 2 min. The subcellular localization of *OsGRP3* was observed by a Leica SP8 STED laser scanning confocal microscope (Leica, Germany).

### RNA-Sequencing Analysis

Total RNAs were extracted from the leaf tissues of *OsGRP3*^*OE*^, *OsGRP3*^*KD*^, and non-transgenic (NT) plants that were grown under normal soil conditions for 4 weeks using the RNeasy plant mini kit (Qiagen, USA) according to the manufacturer’s instruction. cDNA libraries were prepared using the TruSeq RNA Sample Prep kit (v2) (Macrogen, Korea). Single-end sequences were obtained using IRGSP (v 1.0) and raw sequence reads were trimmed to remove adaptor sequence, and those with a quality lower than Q20 were removed using the Trimmomatic 0.32 software (Bolger et al. 2014). To map the reads to reference genome, all reads were assembled with annotated genes from the Rap-DB database [http://rapdb.dna.affrc.go.jp; IRGSP (v 1.0)] using TopHat software (https://ccb.jhu.edu/software/tophat/index.shtml). After mapping reads to a reference genome, differentially expressed genes (DEGs) were selected using a cut-off change of at least 2-fold change and Student’s *t*-test (*P* < 0.05). The selected DEGs were grouped by hierarchical clustering analysis (Complete Linkage).

### RNA-IP Analysis

The RNA-IP analysis was consulted as previously described with minor modifications (Keene et al. 2006; Peritz et al. 2006). Leaf tissues of 14-day-old rice seedlings were homogenized with liquid nitrogen, and the ground powder was incubated with polysome lysis buffer [100 mM KCl, 5 mM MgCl_2_, 10 mM HEPES pH 7.0, 0.5% Nonidet P-40, 1 mM DTT, RNase Out RNase inhibitor (Invitrogen, USA), 2 mM vanadyl ribonucleoside complexes solution (Sigma-Aldrich, USA), protease inhibitor cocktail (Roche, Switzerland)] for 20 min. The supernatant was separated from the crude extracts by centrifuging at 16,000 g for 20 min at 4 °C. After quantification of the soluble proteins by Bradford assay kit (Bio-rad, USA), 1 mg aliquot of the lysates was used for further analysis. Before the immunoprecipitation step, the lysates were pre-cleared with protein A-agarose beads equilibrated in lysis buffer containing 1 mg ml^− 1^ BSA (Sigma, USA) by rotating at 4 °C for 2 h. The aliquot of the pre-cleared lysate was stored and used as a control for total input of RNAs. After pre-clearing, the lysates were incubated with indicated antibodies at 4 °C for 6 h. The protein-RNA complexes were then precipitated using protein A-agarose beads. The beads were washed four times with 1st washing buffer [100 mM KCl, 5 mM MgCl_2_, 10 mM HEPES pH 7.0, 0.5% Nonidet P-40, 1 mM DTT] and additional four times with 2nd washing buffer [100 mM KCl, 5 mM MgCl_2_, 10 mM HEPES pH 7.0, 0.5% Nonidet P-40, 1 mM DTT, 1 M urea]. RNAs were eluted from the beads with the polysome lysis buffer containing 0.1% SDS and 30 μg of proteinase K at 50 °C for 30 min. Immunoprecipitated RNAs were purified using phenol/chloroform extraction and ethanol precipitation. Then, 0.01% of input RNAs were used to determine the relative enrichment of RNAs after immunoprecipitation.

### Measurements of mRNA Half-Lives

The half-life of mRNA was determined in rice plants as previously described (Lidder et al. 2005; Park et al. 2012). Briefly, two-week-old rice seedlings were transferred soil to tap water for 2 days. After adaptation, the rice seedlings were treated with 1 mM of cordycepin (3′-deoxyadenosine) (Sigma, USA) through roots uptake for 30 min. After the treatment, the leaf tissues were harvested at indicated time points. RNA extraction, first-strand synthesis, and qRT-PCR were performed as described in “RNA Isolation and Quantitative Real-Time PCR Analysis.” Half-lives were calculated using Sigma plot software (Sigma Plot v10.0; http://www.systat.com).

### Drought Stress Treatments and Tolerance Evaluation

Non-Transgenic (NT) (*O. sativa* cv. Nakdong for *OsGRP3*^*OE*^ and *O. sativa* cv. Dongjin for *OsGRP3*^*KD*^), *OsGRP3*^*OE*^ and *OsGRP3*^*KD*^ plants were sown on MS solid medium and incubated in a dark growth chamber for 4 days at 28 °C. Seedlings were then transferred to a growth chamber with a light and dark cycle of 16 h light/8 h dark and grown for one additional day before transplanting into soil. Thirty plants from each line were transplanted into ten soil pots (4 × 4 × 6 cm: three plants per pot) within a container (59 × 38.5 × 15 cm) and grown for additional 4 weeks in a greenhouse (16 h light/ 8 h dark cycle) at 30 °C. Drought stress was imposed by sequentially withholding water for 3 days and re-watering for 10 days (Fig. 2). In case of air-drying drought treatment (Figs. 1d, 6, and 7), whole plants were exposed to air by removing the plants from their pots for several hours. Drought-induced symptoms were monitored by imaging transgenic and NT plants at the indicated time points after the drought treatments using a NEX-5 N camera (Sony, Japan). The soil moisture content was measured using the SM150 Soil Moisture Sensor (Delta-T Devices, UK). Transient chlorophyll a fluorescence was measured using the Handy-PEA fluorimeter (Hansatech Instruments, UK). Chlorophyll A fluorescence was measured from the longest leaves of each plant after 1 h of dark adaptation to ensure sufficient opening of the reaction center. Measurement was performed at apex, middle, and base regions of leaves using the Handy-PEA fluorimeter. Thirty measurements per line were averaged using the HANDY-PEA software (version 1.31).

### Histochemical Staining and Measurement of H_2_O_2_

Accumulation of H_2_O_2_ was examined based on histochemical staining by 3, 3′-diaminobenzidine (DAB) as described before (Liu et al. 2012). NT and transgenic leaves of 2 weeks old plants were air-dried for different time intervals (1, 2, 3 h). These leaves were vacuum infiltrated into 1 mg/ml fresh DAB solution (pH 3.8) prepared in 10 mM phosphate buffer (pH 7.8) and incubated in dark cabinet overnight. The stained leaves were then fixed with a solution of 3:1:1 ethanol:lactic acid: glycerol and photographed. The H_2_O_2_ level was measured as described before (Kim et al. 2007a). The 2 weeks old plants (0.3 g fresh weight) were ground in 50 mM phosphate buffer (pH 6.8), and the homogenate was centrifuged at 6000 g for 25 min. To determine the H_2_O_2_ level, 3 ml of the extracted solution was mixed with 1 ml of 0.1% titanium chloride in 20% (v/v) H_2_O_2_, and the mixture was then centrifuged at 6000 g for 15 min. The absorbance of the supernatant was measured at 410 nm, and the H_2_O_2_ level was calculated based on the standard curve generated using authentic H_2_O_2._

### Accession Numbers

Genes from this article can be found in the National Center for Biotechnology Information (http://www.ncbi.nlm.nih.gov/) under the following accession numbers: GSE151351 (RNA-seq), *GRP3* (Os03g0670700), *PR5* (Os12g0629700), *DCP1* (Os12g0156400), *PABP8* (Os09g0115400), *LEA17* (Os03g0322900), *FX1* (Os05g0369900), *POX1* (Os01g026330), *MT1d* (Os12g0571100), *CCD1* (Os08g0371200), *DOPA* (Os01g0878900), *LRR* (Os01g0162300), *LOX* (Os10g0361000), *SAM1*(Os01g0293000).

## Supplementary Information

**Additional file 1: Table S1**. List of primers used in this study

**Additional file 2: Table S2**. List of up- and down-regulated genes in *OsGRP3*^*OE*^ transgenic plants compared to non-transgenic plants

**Additional file 3: Table S3**. Up-regulated genes in *OsGRP3*^*OE*^ transgenic rice plants in comparison with non-transgenic plants

**Additional file 4: Table S4**. List of up- and down-regulated genes in *OsGRP3*^*KD*^ transgenic plants compared to non-transgenic plants

**Additional file 5: Table S5**. Analysis of half-life in non-transgenic, *OsGRP3*^*OE,*^ and *OsGRP3*^*KD*^ transgenic plants

**Additional file 6: Fig. S1**. Phylogenetic analysis of *OsGRP* genes. **Fig. S2**. Expression levels of *OsGRP3* in various rice tissues at different developmental stages. **Fig. S3**. Expression patterns of *OsGRPs* under drought conditions. **Fig. S4**. Drought tolerance of *OsGRP3*^*OE*^ plants. **Fig. S5**. Drought tolerance of *OsGRP3-GFP*^*OE*^ and *MYC*-*OsGRP3*^*OE*^ plants and subcellular localization of OsGRP3 in leaves. **Fig. S6**. Effect of ABA treatments on subcellular localization of OsGRP3. **Fig. S7**. Effect of mannitol treatments on subcellular localization of OsGRP3 in rice protoplasts. **Fig. S8**. Effect of heat treatments on subcellular localization of OsGRP3 in rice protoplasts. **Fig. S9**. Effects of *OsGRP3* on the stability of *DOPA* and *LOX* transcripts. **Fig. S10**. Expression patterns of *OsGRP3*, *DOPA,* and *LOX* under drought conditions

## Data Availability

The datasets supporting the conclusions of this article are provided within the article and its additional files.

## Abbreviations

GRPs: Glycine-rich RNA binding proteins
RRM: RNA-recognition motif
GA: Gibberellin
mRNPs: messenger ribonucleoprotein complexes
DCP1: DECAPPING 1
PABP8: POLY (A) BINDING PROTEIN 8
PR: Pathogenesis-related
LEA17: LATE EMBRYOGENESIS ABUNDANT PROTEIN 17
FX1: EXOCYST SUBUNIT EXO70 FAMILY PROTEIN FX1
POX1: PEROXIDASE 1
MT1d: METALLOTHIONEIN 1d
CCD1: CAROTENOID 9, 10-CLEAVAGE DIOXYGENASE 1
DOPA: 4,5-DOPA DIOXYGENASE EXTRADIOL-LIKE PROTEIN
LRR: LEUCINE-RICH REPEAT DOMAIN CONTAINING PROTEIN
LOX: LIPOXYGENASE
SAM1: S-ADENOSYLMETHOININE SYNTHETASE 1
ROS: reactive oxygen species
PM1: PLASMA MEMBRANE PROTEIN 1

## References

[CR1] Ali S, Ganai BA, Kamili AN, Bhat AA, Mir ZA, Bhat JA, Tyagi A, Islam ST, Mushtaq M, Yadav P, Rawat S, Grover A (2018). Pathogenesis-related proteins and peptides as promising tools for engineering plants with multiple stress tolerance. Microbiol Res.

[CR2] Auldridge ME, Block A, Vogel JT, Dabney-Smith C, Mila I, Bouzayen M, Magallanes-Lundback M, DellaPenna D, McCarty DR, Klee HJ (2006). Characterization of three members of the Arabidopsis carotenoid cleavage dioxygenase family demonstrates the divergent roles of this multifunctional enzyme family. Plant J.

[CR3] Bogamuwa S, Jang JC (2013). The Arabidopsis tandem CCCH zinc finger proteins AtTZF4, 5 and 6 are involved in light-, abscisic acid- and gibberellic acid-mediated regulation of seed germination. Plant Cell Environ.

[CR4] Bolger AM, Lohse M, Usadel B (2014). Trimmomatic: a flexible trimmer for Illumina sequence data. Bioinformatics.

[CR5] Chen X, Q-c Z, Lu X-p, Yu D-q, W-z L (2010). Characterization and expression analysis of four glycine-rich RNA-binding proteins involved in osmotic response in tobacco (*Nicotiana tabacum cv. Xanthi*). Agric Sci China.

[CR6] Ciuzan O, Hancock J, Pamfil D, Wilson I, Ladomery M (2015). The evolutionarily conserved multifunctional glycine-rich RNA-binding proteins play key roles in development and stress adaptation. Physiol Plant.

[CR7] Condit CM, Meagher RB (1986). A gene encoding a novel glycine-rich structural protein of petunia. Nature.

[CR8] Duan J, Cai W (2012). *OsLEA3-2*, an abiotic stress induced gene of rice plays a key role in salt and drought tolerance. PLoS One.

[CR9] Fusaro AF, Bocca SN, Ramos RL, Barroco RM, Magioli C, Jorge VC, Coutinho TC, Rangel-Lima CM, De Rycke R, Inze D, Engler G, Sachetto-Martins G (2007). AtGRP2, a cold-induced nucleo-cytoplasmic RNA-binding protein, has a role in flower and seed development. Planta.

[CR10] Gill SS, Tuteja N (2010). Reactive oxygen species and antioxidant machinery in abiotic stress tolerance in crop plants. Plant Physiol Biochem.

[CR11] Grennan AK (2011). Metallothioneins, a diverse protein family. Plant Physiol.

[CR12] Hackmann C, Korneli C, Kutyniok M, Koster T, Wiedenlubbert M, Muller C, Staiger D (2014). Salicylic acid-dependent and -independent impact of an RNA-binding protein on plant immunity. Plant Cell Environ.

[CR13] Han M, Kim CY, Lee J, Lee SK, Jeon JS (2014). *OsWRKY42* represses *OsMT1d* and induces reactive oxygen species and leaf senescence in rice. Mol Cell.

[CR14] Jan A, Maruyama K, Todaka D, Kidokoro S, Abo M, Yoshimura E, Shinozaki K, Nakashima K, Yamaguchi-Shinozaki K (2013). OsTZF1, a CCCH-tandem zinc finger protein, confers delayed senescence and stress tolerance in rice by regulating stress-related genes. Plant Physiol.

[CR15] Jang IC, Choi WB, Lee KH, Song SI, Nahm BH, Kim JK (2002). High-level and ubiquitous expression of the rice cytochrome c gene *OsCc1* and its promoter activity in transgenic plants provides a useful promoter for transgenesis of monocots. Plant Physiol.

[CR16] Jang I-C, Nahm BH, Kim J-K (1999). Subcellular targeting of green fluorescent protein to plastids in transgenic rice plants provides a high-level expression system. Mol Breed.

[CR17] Jeong JS, Kim YS, Baek KH, Jung H, Ha SH, Do Choi Y, Kim M, Reuzeau C, Kim JK (2010). Root-specific expression of *OsNAC10* improves drought tolerance and grain yield in rice under field drought conditions. Plant Physiol.

[CR18] Kang H, Park SJ, Kwak KJ (2013). Plant RNA chaperones in stress response. Trends Plant Sci.

[CR19] Kaur R, Chakraborty A, Bhunia RK, Sen SK, Ghosh AK (2017). Tolerance to soil water stress by *Oryza sativa* cv. IR20 was improved by expression of *Wsi18* gene locus from *Oryza nivara*. Biol Plant.

[CR20] Kedersha N, Stoecklin G, Ayodele M, Yacono P, Lykke-Andersen J, Fritzler MJ, Scheuner D, Kaufman RJ, Golan DE, Anderson P (2005). Stress granules and processing bodies are dynamically linked sites of mRNP remodeling. J Cell Biol.

[CR21] Keene JD, Komisarow JM, Friedersdorf MB (2006). RIP-Chip: the isolation and identification of mRNAs, microRNAs and protein components of ribonucleoprotein complexes from cell extracts. Nat Protoc.

[CR22] Kim JS, Jung HJ, Lee HJ, Kim KA, Goh C-H, Woo Y, Oh SH, Han YS, Kang H (2008). Glycine-rich RNA-binding protein7 affects abiotic stress responses by regulating stomata opening and closing in *Arabidopsis thaliana*. Plant J.

[CR23] Kim JS, Park SJ, Kwak KJ, Kim YO, Kim JY, Song J, Jang B, Jung CH, Kang H (2007). Cold shock domain proteins and glycine-rich RNA-binding proteins from Arabidopsis thaliana can promote the cold adaptation process in *Escherichia coli*. Nucleic Acids Res.

[CR24] Kim JY, Kim WY, Kwak KJ, Oh SH, Han YS, Kang H (2010). Glycine-rich RNA-binding proteins are functionally conserved in *Arabidopsis thaliana* and *Oryza sativa* during cold adaptation process. J Exp Bot.

[CR25] Kim JY, Park SJ, Jang B, Jung CH, Ahn SJ, Goh CH, Cho K, Han O, Kang H (2007). Functional characterization of a glycine-rich RNA-binding protein 2 in *Arabidopsis thaliana* under abiotic stress conditions. Plant J.

[CR26] Kim YO, Pan S, Jung CH, Kang H (2007). A zinc finger-containing glycine-rich RNA-binding protein, atRZ-1a, has a negative impact on seed germination and seedling growth of Arabidopsis thaliana under salt or drought stress conditions. Plant Cell Physiol.

[CR27] Kumar G, Kushwaha HR, Panjabi-Sabharwal V, Kumari S, Joshi R, Karan R, Mittal S, Pareek SLS, Pareek A. Clustered metallothionein genes are co-regulated in rice and ectopic expression of OsMT1e-P confeavenging. BMC Plant Biol 2012;12:107. 10.1186/1471-2229-12-107 10.1186/1471-2229-12-107 PMC3491035 22780875

[CR28] Kwak KJ, Park SJ, Han JH, Kim MK, Oh SH, Han YS, Kang H (2011). Structural determinants crucial to the RNA chaperone activity of glycine-rich RNA-binding proteins 4 and 7 in Arabidopsis thaliana during the cold adaptation process. J Exp Bot.

[CR29] Lee DK, Kim HI, Jang G, Chung PJ, Jeong JS, Kim YS, Bang SW, Jung H, Choi YD, Kim JK (2015). The NF-YA transcription factor OsNF-YA7 confers drought stress tolerance of rice in an abscisic acid independent manner. Plant Sci.

[CR30] Li R, Liu C, Zhao R, Wang L, Chen L, Yu W, Zhang S, Sheng J, Shen L (2019). CRISPR/Cas9-mediated *SlNPR1* mutagenesis reduces tomato plant drought tolerance. BMC Plant Biol.

[CR31] Liang Y, Kang K, Gan L, Ning SB, Xiong JY, Song SY, Xi LZ, Lai SY, Yin YT, Gu JW, Xiang J, Li SS, Wang BS, Li MT (2019). Drought-responsive genes, late embryogenesis abundant group3 (LEA3) and vicinal oxygen chelate, function in lipid accumulation in Brassica napus and Arabidopsis mainly via enhancing photosynthetic efficiency and reducing ROS. Plant Biotechnol J.

[CR32] Lidder P, Gutierrez RA, Salome PA, McClung CR, Green PJ (2005). Circadian control of messenger RNA stability. Association with a sequence-specific messenger RNA decay pathway. Plant Physiol.

[CR33] Liu WX, Zhang FC, Zhang WZ, Song LF, Wu WH, Chen YF (2013). *Arabidopsis* Di19 functions as a transcription factor and modulates *PR1*, *PR2*, and *PR5* expression in response to drought stress. Mol Plant.

[CR34] Liu Z, Zhang Z, Faris JD, Oliver RP, Syme R, McDonald MC, McDonald BA, Solomon PS, Lu S, Shelver WL, Xu S, Friesen TL (2012). The cysteine rich necrotrophic effector SnTox1 produced by Stagonospora nodorum triggers susceptibility of wheat lines harboring Snn1. PLoS Pathog.

[CR35] Liu ZZ, Wang JL, Huang X, Xu WH, Liu ZM, Fang RX (2003). The promoter of a rice glycine-rich protein gene, *Osgrp-2*, confers vascular-specific expression in transgenic plants. Planta.

[CR36] Löhr B, Streitner C, Steffen A, Lange T, Staiger D (2014). A glycine-rich RNA-binding protein affects gibberellin biosynthesis in *Arabidopsis*. Mol Biol Rep.

[CR37] Lorkovic ZJ (2009). Role of plant RNA-binding proteins in development, stress response and genome organization. Trends Plant Sci.

[CR38] Lummer M, Humpert F, Steuwe C, Caesar K, Schuttpelz M, Sauer M, Staiger D (2011). Reversible photoswitchable DRONPA-s monitors nucleocytoplasmic transport of an RNA-binding protein in transgenic plants. Traffic.

[CR39] Magdalena C, Michal R (2018). Plant glycine-rich proteins in stress response: an emerging, still prospective story. Front Plant Sci.

[CR40] Meyer K, Köster T, Nolte C, Weinholdt C, Lewinski M, Grosse I, Staiger D (2017). Adaptation of iCLIP to plants determines the binding landscape of the clock-regulated RNA-binding protein *At*GRP7. Genome Biol.

[CR41] Nisar N, Li L, Lu S, Khin Nay C, Pogson Barry J (2015). Carotenoid metabolism in plants. Mol Plant.

[CR42] Park AR, Cho SK, Yun UJ, Jin MY, Lee SH, Sachetto-Martins G, Park OK (2001). Interaction of the Arabidopsis receptor protein kinase Wak1 with a glycine-rich protein, AtGRP-3. J Biol Chem.

[CR43] Park SH, Chung PJ, Juntawong P, Bailey-Serres J, Kim YS, Jung H, Bang SW, Kim YK, Do Choi Y, Kim JK (2012). Posttranscriptional control of photosynthetic mRNA decay under stress conditions requires 3′ and 5′ untranslated regions and correlates with differential polysome association in rice. Plant Physiol.

[CR44] Peritz T, Zeng F, Kannanayakal TJ, Kilk K, Eiriksdottir E, Langel U, Eberwine J (2006). Immunoprecipitation of mRNA-protein complexes. Nat Protoc.

[CR45] Pinheiro C, Chaves MM (2010). Photosynthesis and drought: can we make metabolic connections from available data?. J Exp Bot.

[CR46] Pomeranz MC, Hah C, Lin PC, Kang SG, Finer JJ, Blackshear PJ, Jang JC (2010). The Arabidopsis tandem zinc finger protein AtTZF1 traffics between the nucleus and cytoplasmic foci and binds both DNA and RNA. Plant Physiol.

[CR47] Qiu Y, Yu D (2009). Over-expression of the stress-induced *OsWRKY45* enhances disease resistance and drought tolerance in *Arabidopsis*. Environ Exp Bot.

[CR48] Ringli C, Hauf G, Keller B (2001). Hydrophobic interactions of the structural protein GRP1.8 in the cell wall of protoxylem elements. Plant Physiol.

[CR49] Schmidt F, Marnef A, Cheung MK, Wilson I, Hancock J, Staiger D, Ladomery M (2010). A proteomic analysis of oligo(dT)-bound mRNP containing oxidative stress-induced Arabidopsis thaliana RNA-binding proteins ATGRP7 and ATGRP8. Mol Biol Rep.

[CR50] Schoning JC, Streitner C, Meyer IM, Gao Y, Staiger D (2008). Reciprocal regulation of glycine-rich RNA-binding proteins via an interlocked feedback loop coupling alternative splicing to nonsense-mediated decay in Arabidopsis. Nucleic Acids Res.

[CR51] Seo Y-S, Chern M, Bartley LE, Han M, Jung K-H, Lee I, Walia H, Richter T, Xu X, Cao P, Bai W, Ramanan R, Amonpant F, Arul L, Canlas PE, Ruan R, Park C-J, Chen X, Hwang S, Jeon J-S, Ronald PC (2011). Towards establishment of a rice stress response interactome. PLoS Genet.

[CR52] Shim JS, Oh N, Chung PJ, Kim YS, Choi YD, Kim J-K (2018) Overexpression of *OsNAC14* improves drought tolerance in rice. Front Plant Sci 9. 10.3389/fpls.2018.00310 10.3389/fpls.2018.00310 PMC5855183 29593766

[CR53] Streitner C, Hennig L, Korneli C, Staiger D (2010). Global transcript profiling of transgenic plants constitutively overexpressing the RNA-binding protein *At*GRP7. BMC Plant Biol.

[CR54] Streitner C, Koster T, Simpson CG, Shaw P, Danisman S, Brown JW, Staiger D (2012). An hnRNP-like RNA-binding protein affects alternative splicing by *in vivo* interaction with transcripts in *Arabidopsis thaliana*. Nucleic Acids Res.

[CR55] Thanin C, Julia B-S (2018). Polysomes, stress granules, and processing bodies: a dynamic triumvirate controlling cytoplasmic mRNA fate and function. Plant Physiol.

[CR56] Walter MH, Strack D (2011). Carotenoids and their cleavage products: biosynthesis and functions. Nat Prod Rep.

[CR57] Wang B, Wang G, Shen F, Zhu S (2018) A glycine-rich RNA-binding protein, CsGR-RBP3, is involved in defense responses against cold stress in harvested cucumber (Cucumis sativus L.) fruit. Front Plant Sci 9:540. 10.3389/fpls.2018.00540 10.3389/fpls.2018.00540 PMC5925850 29740470

[CR58] Wolozin B (2012). Regulated protein aggregation: stress granules and neurodegeneration. Mol Neurodegener.

[CR59] Wu J, Kim SG, Kang KY, Kim J-G, Park S-R, Gupta R, Kim YH, Wang Y, Kim ST (2016). Overexpression of a pathogenesis-related protein 10 enhances biotic and abiotic stress tolerance in rice. Plant Pathol J.

[CR60] Wu KL, Guo ZJ, Wang HH, Li J (2005). The WRKY family of transcription factors in rice and Arabidopsis and their origins. DNA Res.

[CR61] Xu D, Lei M, Wu R (1995). Expression of the rice *Osgrp1* promoter-Gus reporter gene is specifically associated with cell elongation/expansion and differentiation. Plant Mol Biol.

[CR62] Yang DH, Kwak KJ, Kim MK, Park SJ, Yang KY, Kang H (2014). Expression of Arabidopsis glycine-rich RNA-binding protein AtGRP2 or AtGRP7 improves grain yield of rice (*Oryza sativa*) under drought stress conditions. Plant Sci.

[CR63] Yao L, Cheng X, Gu Z, Huang W, Li S, Wang L, Wang Y-F, Xu P, Ma H, Ge X (2018). The AWPM-19 family protein OsPM1 mediates abscisic acid influx and drought response in rice. Plant Cell.

[CR64] Yao LM, Jiang YN, Lu XX, Wang B, Zhou P, Wu TL. Overexpression of a glycine-rich protein gene in *Lablab purpureus* improves abiotic stress tolerance. Genet Mol Res 2016;15(4). 10.4238/gmr15048064 10.4238/gmr15048063 27813556

[CR65] Yoshida S, Forno AD, Cock HJ, Gomez AK (1976). Laboratory manual for physiological studies of rice.

[CR66] You J, Chan Z (2015). ROS regulation during abiotic stress responses in crop plants. Front Plant Sci.

[CR67] Zhu W, Zhao D-X, Miao Q, Xue T-T, Li X-Z, Zheng C-C (2009). *Arabidopsis thaliana* metallothionein, AtMT2a, mediates ROS balance during oxidative stress. J Plant Biol.

