# Supplementary material for: The Rice GLYCINE-RICH PROTEIN 3 Confers Drought Tolerance by Regulating mRNA Stability of ROS Scavenging-Related Genes
Source: Rice (N Y). 2021 Mar 19;14:31. doi: 10.1186/s12284-021-00473-0 (PMC7979854; doi:10.1186/s12284-021-00473-0)
Supplement: Supplementary file 5 — Additional file 5: Table S5. Analysis of half-life in non-transgenic, OsGRP3OE, and OsGRP3KD transgenic plants [file 12284_2021_473_MOESM5_ESM.pdf]

Table S5. Analysis of half-life in non-trasngenic, *OsGRP3*^{OE} and *OsGRP3*^{KD} transgenic plants.

Gene	Plants	Half-life	Equation		
		min	a	b	Y=aX+b
PR5	NT	115.38	-0.00433	1	y=-0.0043x+1
	OsGRP3^{OE}	187.50	-0.00267	1	y=-0.0026x+1
	OsGRP^{KD}	93.75	-0.00533	1	y=-0.0053x+1
PR5 (Drought)[b]	NT	263.16	-0.00190	1	y=-0.0019x+1
	OsGRP3^{OE}	N.D.[a]	0.00160	1	y=0.0016x+1
	OsGRP^{KD}	238.10	-0.00210	1	y=-0.0021x+1
MT1d	NT	187.50	-0.00267	1	y=-0.0026x+1
	OsGRP3^{OE}	107.14	-0.00467	1	y=-0.0046x+1
	OsGRP^{KD}	250.00	-0.00200	1	y=-0.0020x+1
MT1d (Drought) [b]	NT	N.D.[a]	0.00317	1	y=0.0031x+1
	OsGRP3^{OE}	N.D.[a]	0.00177	1	y=0.0017x+1
	OsGRP^{KD}	N.D.[a]	0.00383	1	y=0.0038x+1
DOPA	NT	306.75	-0.00163	1	y=-0.0016x+1
	OsGRP3^{OE}	409.84	-0.00122	1	y=-0.0013x+1
	OsGRP^{KD}	204.08	-0.00245	1	y=-0.0023x+1
LOX	NT	427.34	-0.00119	1	y=-0.0013x+1
	OsGRP3^{OE}	230.41	-0.00217	1	y=-0.0023x+1
	OsGRP^{KD}	462.96	-0.00098	1	y=-0.0013x+1

[a]Not Determined, half-lives were not determined due to positive value of slope. [b]Stability of RNAs was analyzed under drought conditions.
