# Supplementary material for: The Rice GLYCINE-RICH PROTEIN 3 Confers Drought Tolerance by Regulating mRNA Stability of ROS Scavenging-Related Genes
Source: Rice (N Y). 2021 Mar 19;14:31. doi: 10.1186/s12284-021-00473-0 (PMC7979854; doi:10.1186/s12284-021-00473-0)
Supplement: Supplementary file 6 — Additional file 6: Fig. S1. Phylogenetic analysis of OsGRP genes. Fig. S2. Expression levels of OsGRP3 in various rice tissues at different developmental stages. Fig. S3. Expression patterns of OsGRPs under drought conditions. Fig. S4. Drought tolerance of OsGRP3OE plants. Fig. S5. Drought tolerance of OsGRP3-GFPOE and MYC-OsGRP3OE plants and subcellular localization of OsGRP3 in leaves. Fig. S6. Effect of ABA treatments on subcellular localization of OsGRP3. Fig. S7. Effect of mannitol treatments on subcellular localization of OsGRP3 in rice protoplasts. Fig. S8. Effect of heat treatments on subcellular localization of OsGRP3 in rice protoplasts. Fig. S9. Effects of OsGRP3 on the stability of DOPA and LOX transcripts. Fig. S10. Expression patterns of OsGRP3, DOPA, and LOX under drought conditions [file 12284_2021_473_MOESM6_ESM.pdf]

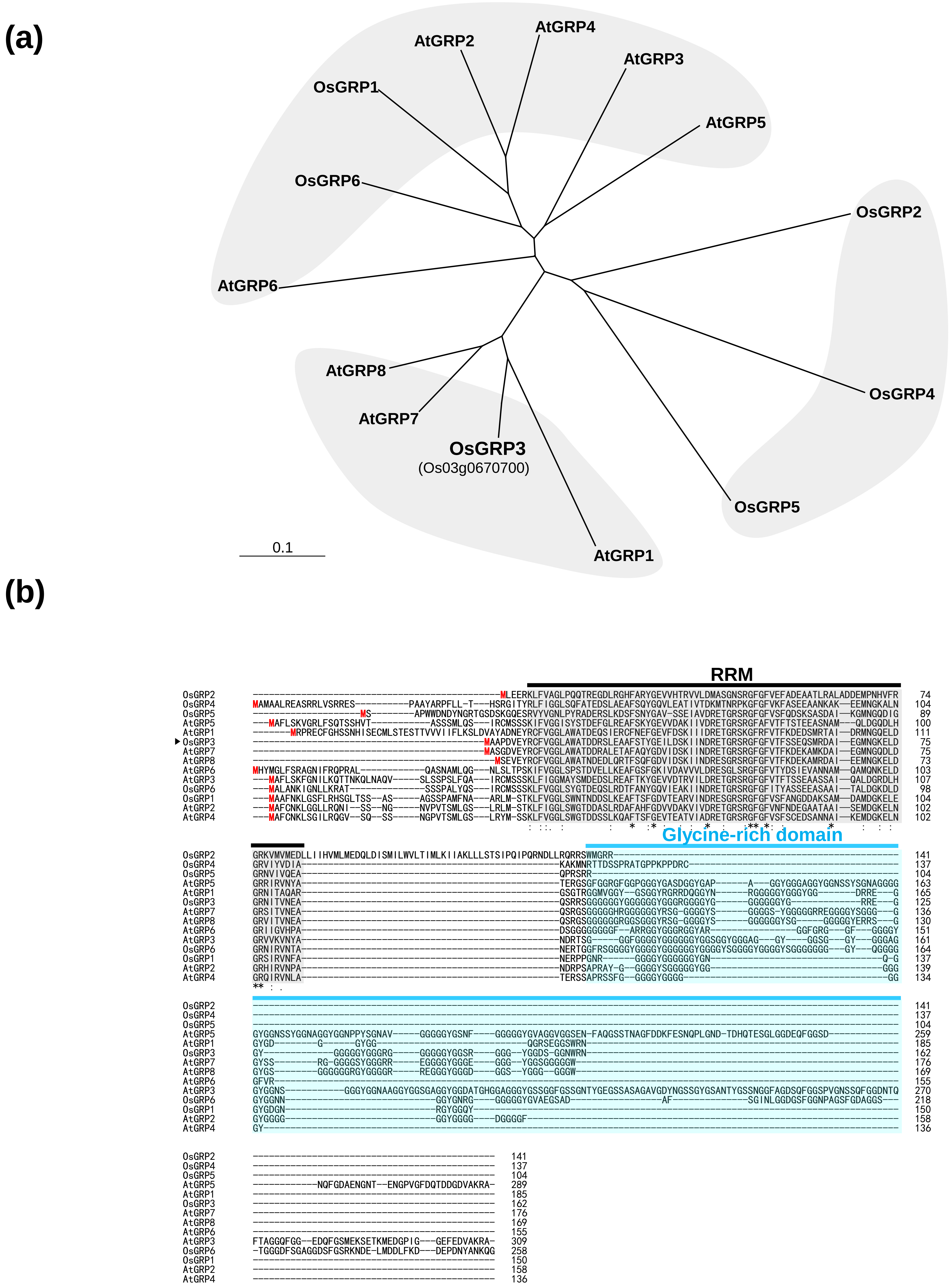


**Figure S1. Phylogenetic analysis of *OsGRP* genes. (a)** Phylogenetic tree was generated using the neighbor-joining method in ClustalW (http://www.clustal.org/clustal2/) using full-length amino acid sequences of Arabidopsis and rice GRP proteins. **(b)** Amino acid alignment of Arabidopsis and Rice GRP proteins. RRM; RNA-Recognition motif.

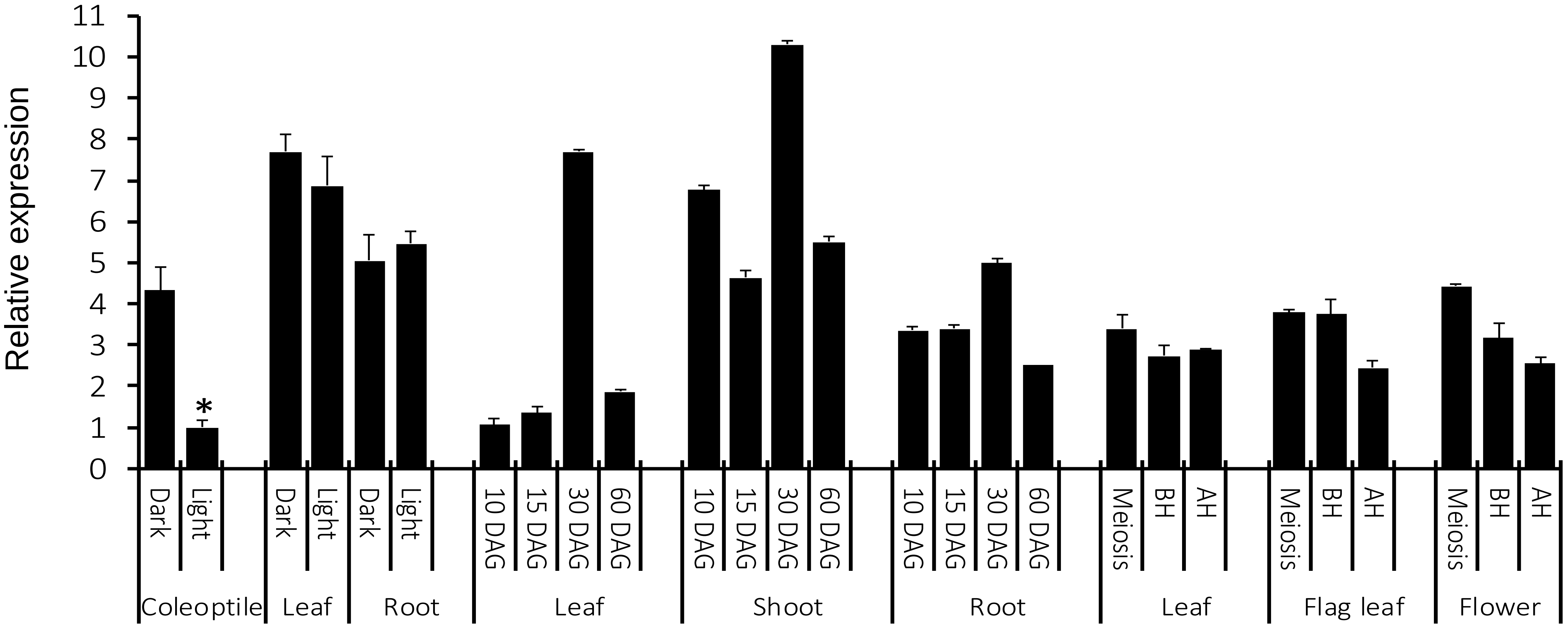


**Figure S2. Expression levels of *OsGRP3* in various rice tissues at different developmental stages.** qRT-PCR analysis was performed using RNA extracted from various rice tissues at different developmental stages (*Oryza sativa*. L. Japonica cv.Ilmi). DAG, day after germination; BH, Before heading; AH, after heading. Rice *UBIQUITIN* (*OsUbi*) was used as an internal control for normalization. Data represent mean value + standard deviation (SD) (n=3). Relative expression value was represented by expression level in Coleoptile (asterisk mark)

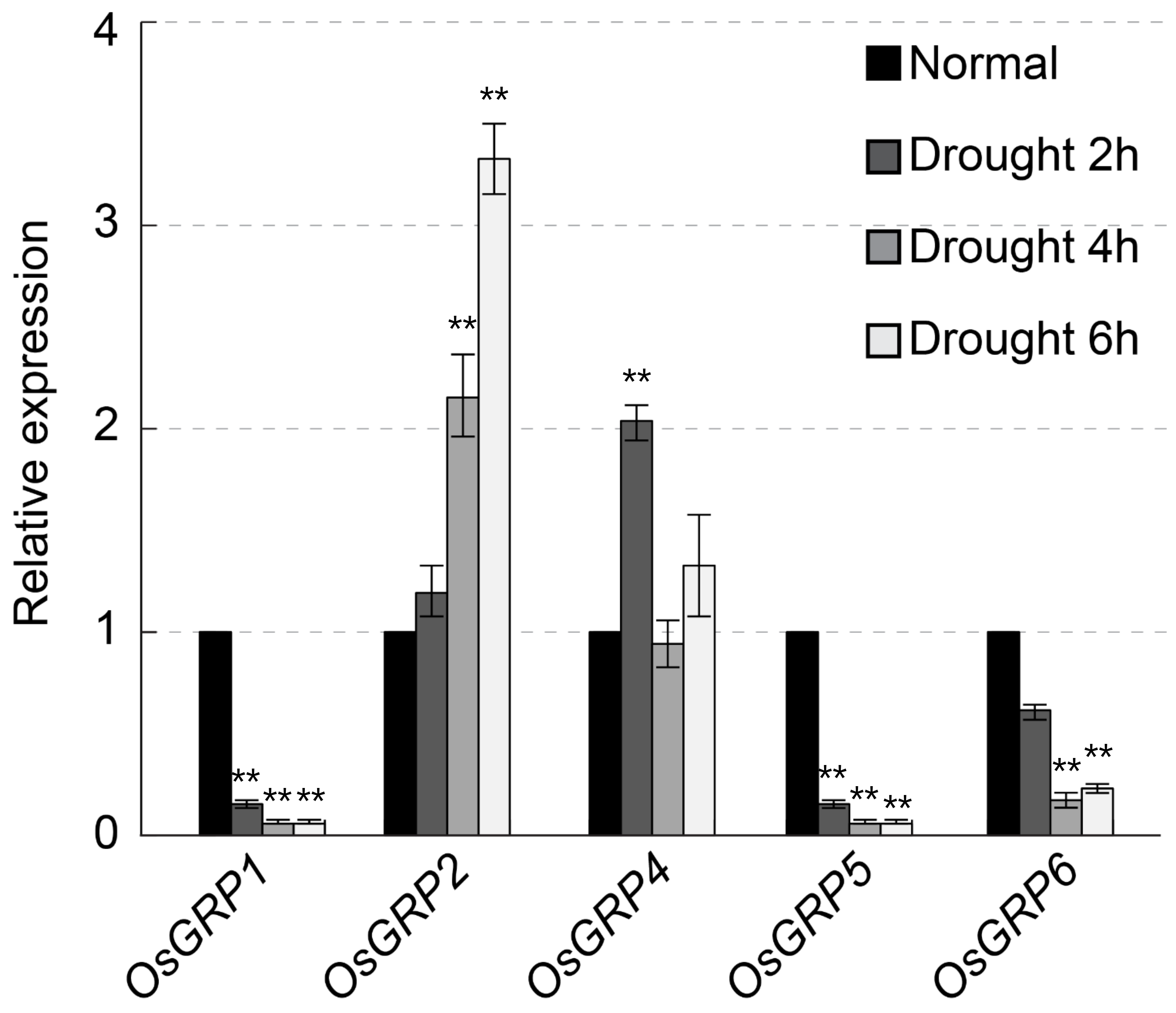


**Figure S3. Expression patterns of *OsGRPs* under drought conditions.** Expression patterns of *OsGRPs* were analyzed by qRT-PCR. Two-week-old rice seedlings were exposed to drought stress and leaves of the treated rice seedlings were harvested at indicated time point after the treatments. Rice *UBIQUITIN* (*OsUbi*) was used as an internal control for normalization. Data represent mean value ± standard deviation (SD) (n=3). Asterisks indicate statistically significant difference compared with normal sample (without drought treatment) as analyzed by one-way ANOVA followed by t -test: **, P  < 0.01.

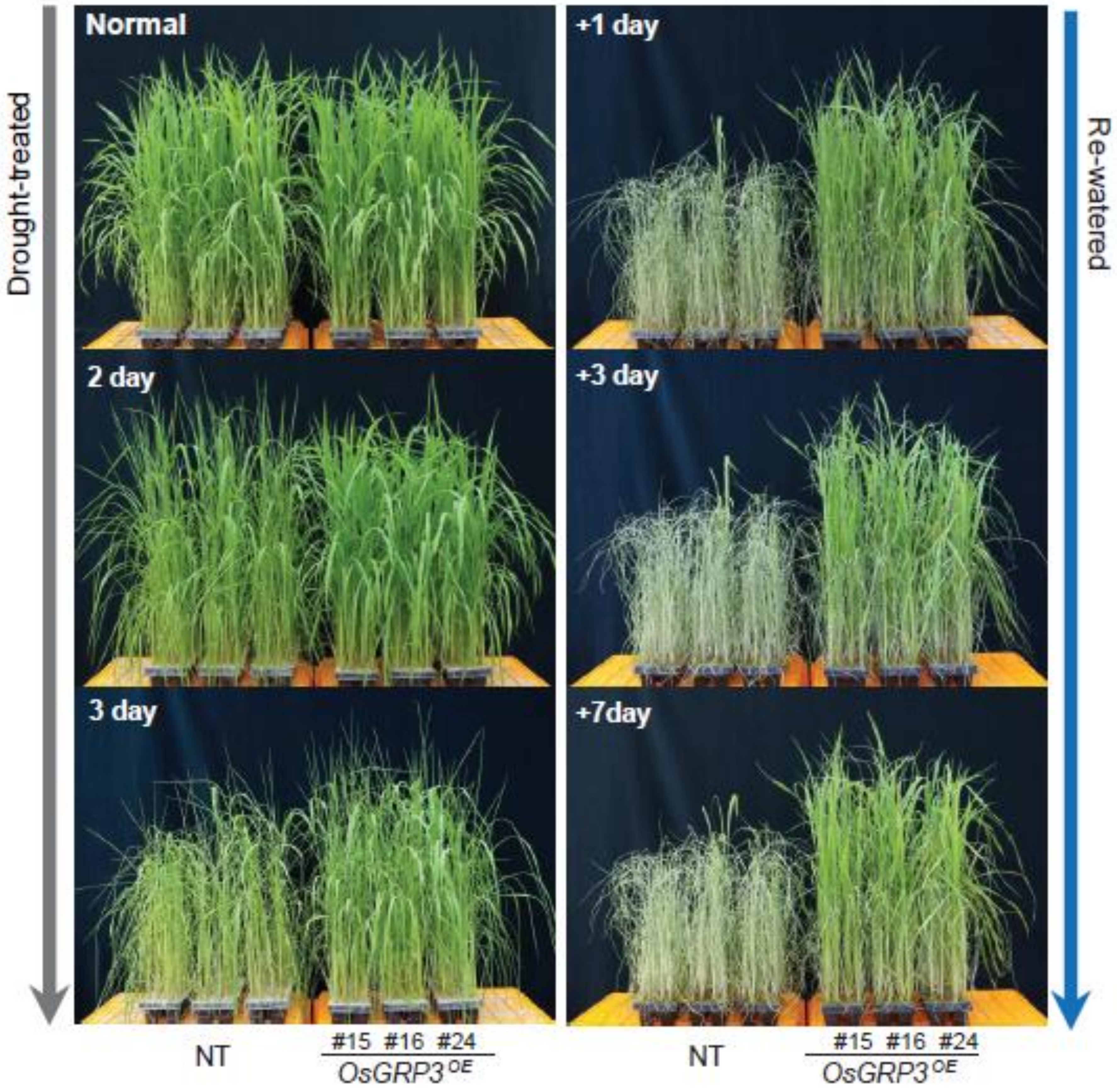


**Figure S4. Drought tolerance of *OsGRP3$^{OE}$* plants.** Four-week-old non-transgenic (NT) and *OsGRP3* overexpressing plants (*OsGRP3$^{OE}$*) were subjected to drought stress by withholding water for 3 days, followed by re-watering for 7 days. The phenotype of tested plants was visualized by taking pictures at indicated time point after drought treatment and re-watering.

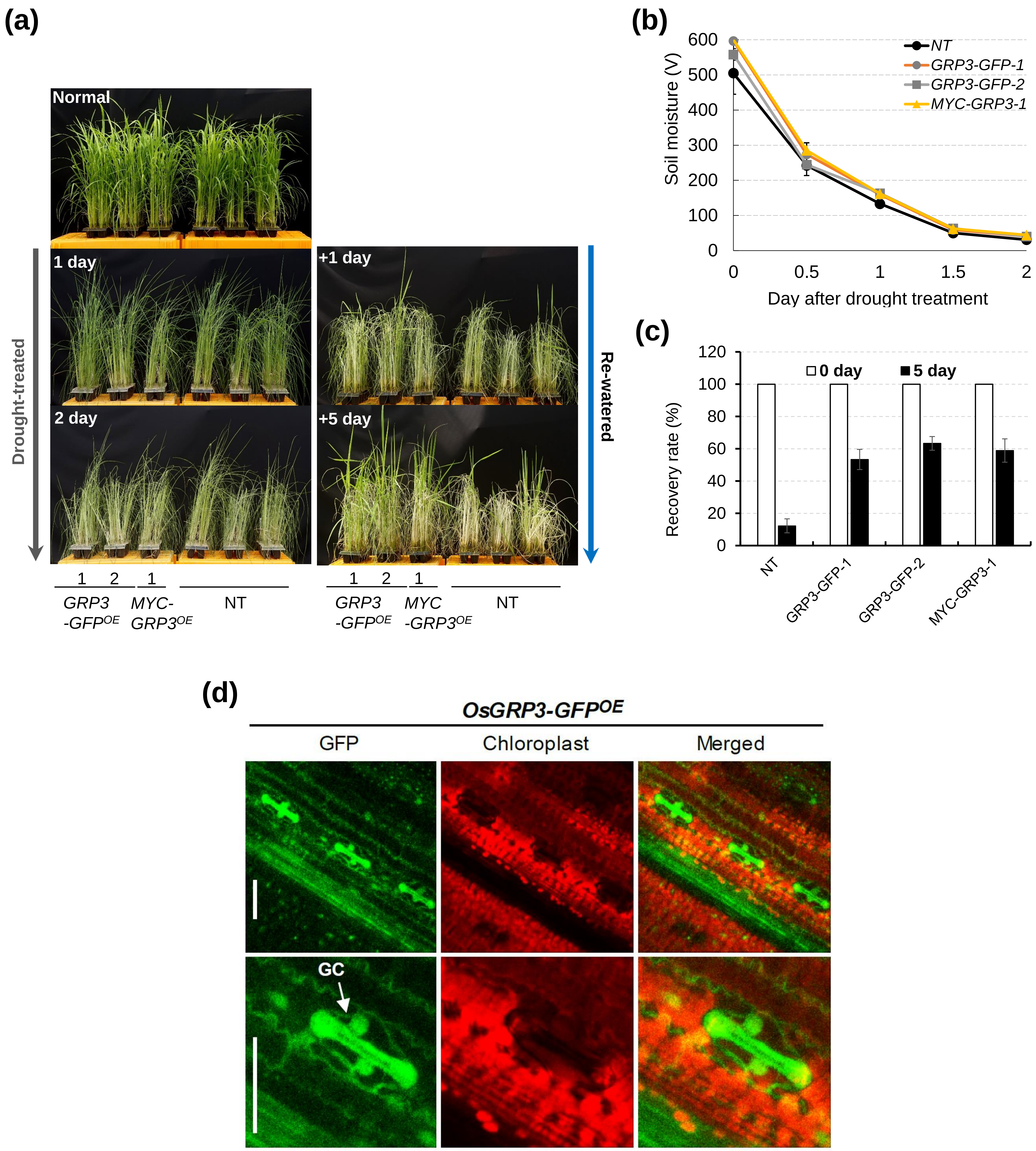


**Figure S5. Drought phenotypes of *OsGRP3-GFP*$^{OE}$ and *MYC-OsGRP3*$^{OE}$ plants and subcellular localization of OsGRP3 in leaves of rice plants. (a)** Four-week-old non-transgenic (NT), *OsGRP3-GFP* (*OsGRP3-GFP*$^{OE}$), or *MYC-OsGRP3* overexpressing plants (*MYC-OsGRP3*$^{OE}$) were subjected to drought stress by withholding water for 2 days, followed by re-watering for 5 days. The phenotype of tested plants was visualized by taking pictures at indicated time point after drought treatment and re-watering. **(b)** Soil moisture content was monitored during drought treatment. Data represent the mean value ± standard deviation (SD) of 30 independent measurements performed at different locations of pots. **(c)** The survival rate of NT and *OsGRP3-GFP*$^{OE}$ transgenic plants was calculated by counting the number of plants recovered from drought stress after re-watering. **(d)** GFP fluorescence and auto-fluorescence of chloroplasts were detected in leaves of *OsCc1::OsGRP3-GFP* (*OsGRP3-GFP*$^{OE}$) using a confocal microscopy. GC: guard cell, Bar= 30 μm.

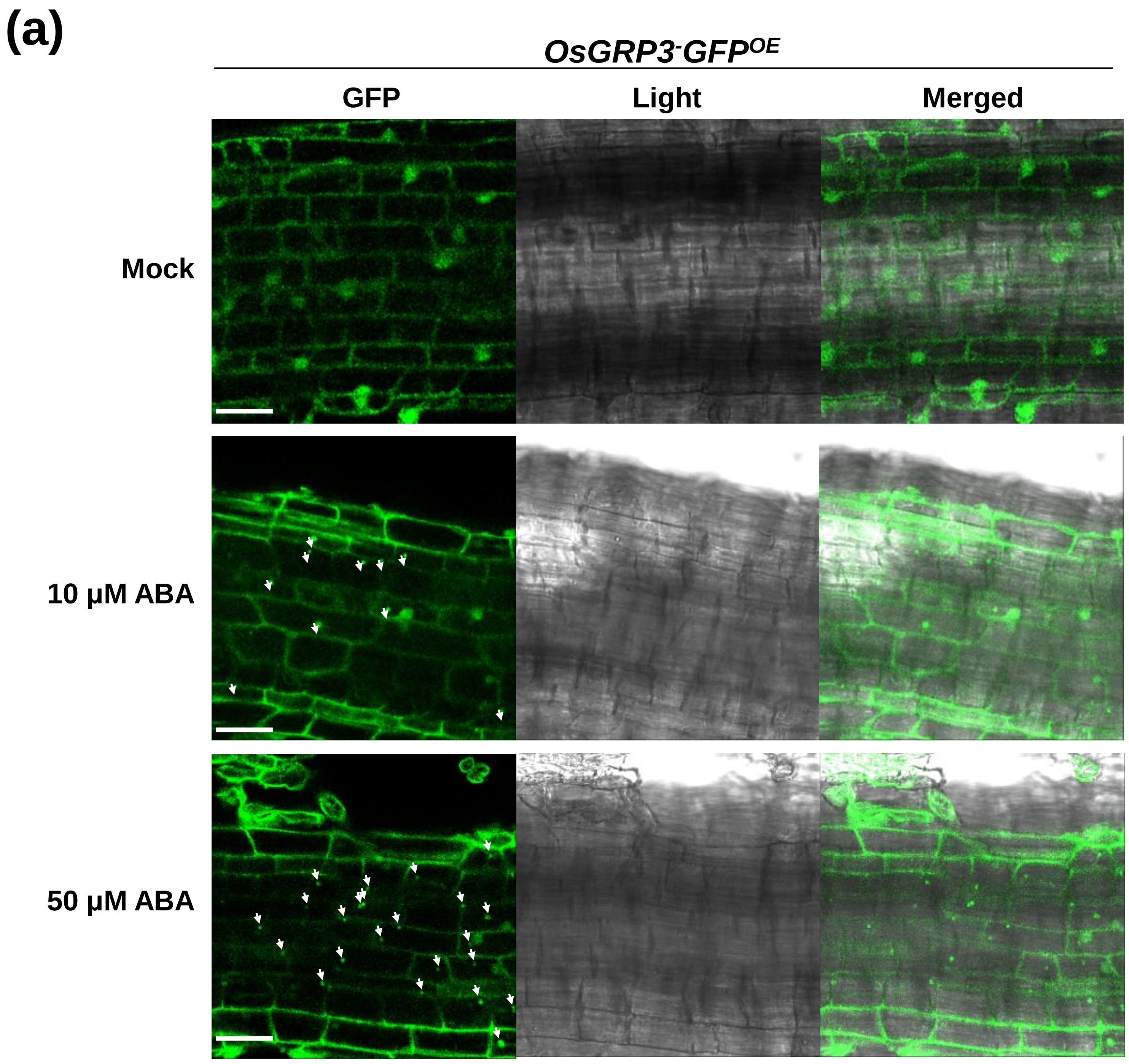


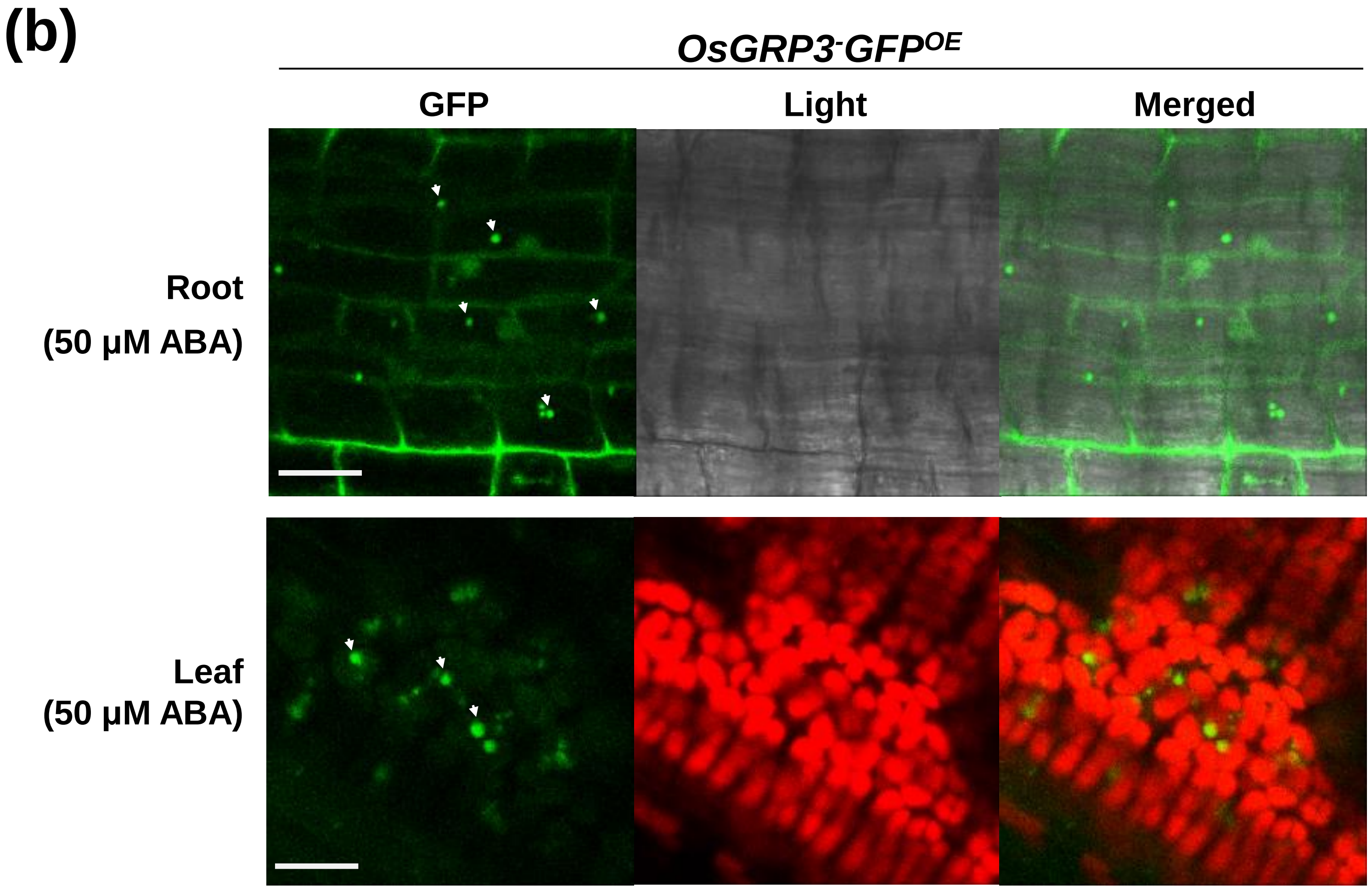


**Figure S6. Effect of ABA treatments on subcellular localization of OsGRP3. (a)** Two-week-old *OsCc1::OsGRP3-GFP* (*OsGRP3-GFP$^{OE}$*) transgenic plants were treated with two different concentrations of ABA (10 μM and 50 μM) for two hours. GFP fluorescence was detected in roots of *OsGRP3-GFP$^{OE}$* transgenic plants using a confocal microscopy. **(b)** Two-week-old *GOS2::OsGRP3-GFP* (*OsGRP3-GFP$^{OE}$*) transgenic plant were treated with 50 μM ABA for two hours. GFP fluorescence was detected in root or leaf **(c)** of *OsGRP3-GFP$^{OE}$* transgenic plants after drought treatments using a confocal microscopy. Arrow heads indicate cytoplasmic foci detected in ABA-treated plants. Bar= 30 μm.

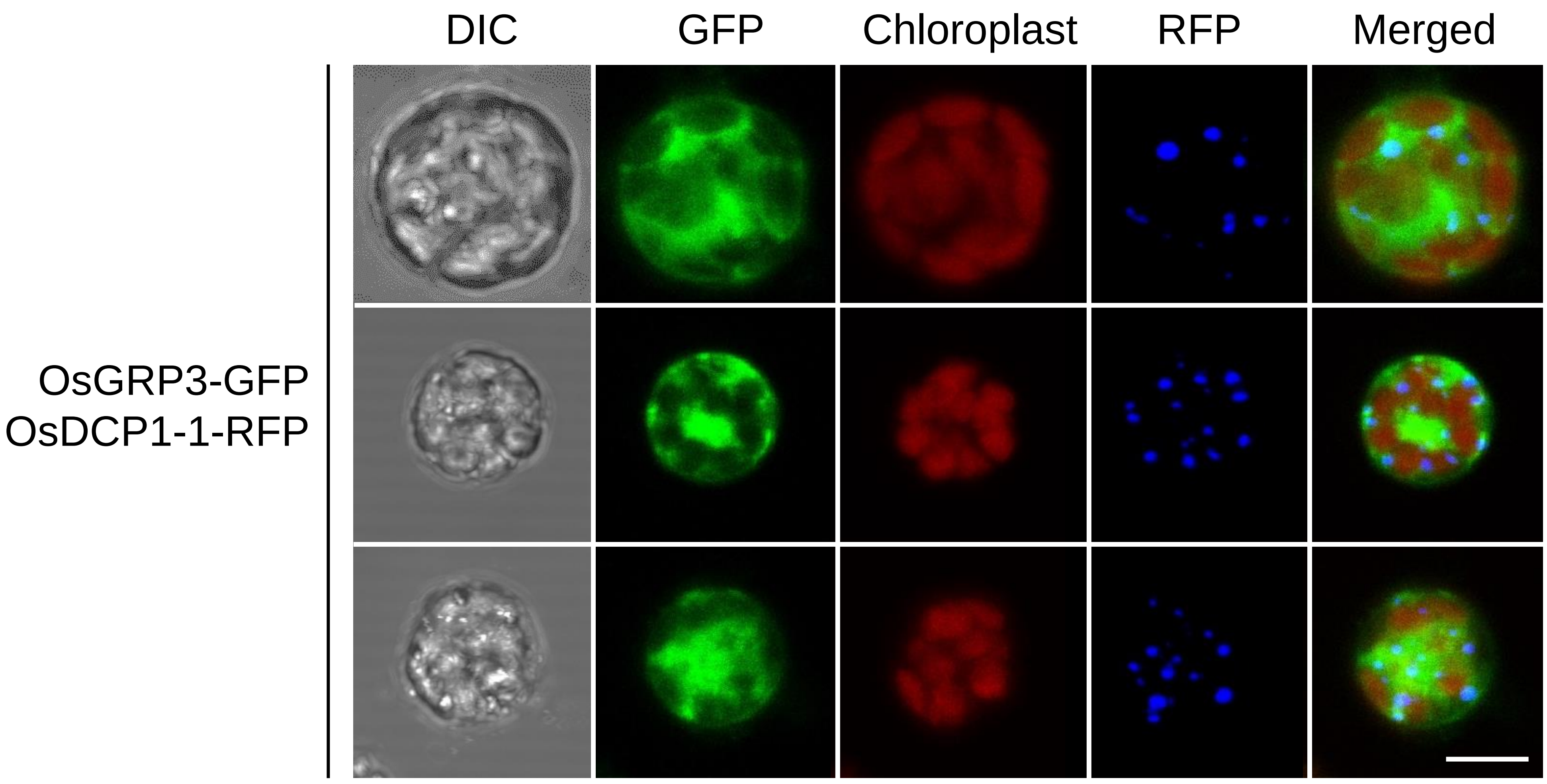


**Figure S7. Effect of mannitol treatments on subcellular localization of OsGRP3 in rice protoplasts.** Rice protoplasts were transformed with constructs expressing OsGRP3-GFP and OsDCP1-RFP. Fluorescence of GFP, RFP and chloroplast were analyzed in the transformed protoplasts 3 hours after 1.5 M mannitol treatments using a confocal microscope. Bar=10 μm.

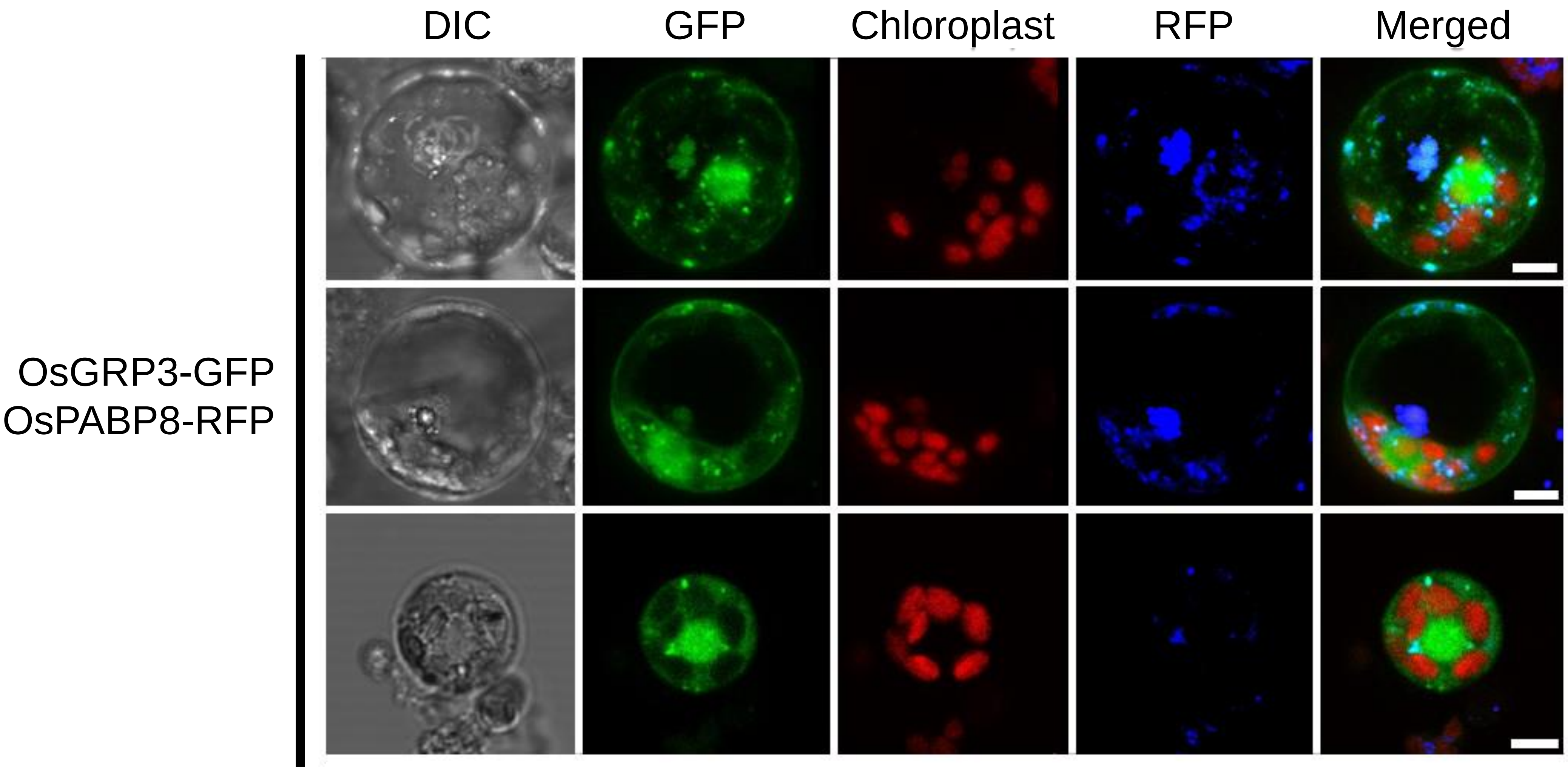


**Figure S8. Effect of heat treatments on subcellular localization of OsGRP3 in rice protoplasts.** Rice protoplasts were transformed with constructs expressing OsGRP3-GFP and OsPABP8-RFP. Fluorescence of GFP, RFP and chloroplast were analyzed in the transformed protoplasts 3 hours after incubation at 42°C using a confocal microscope. Bar=10 μm.

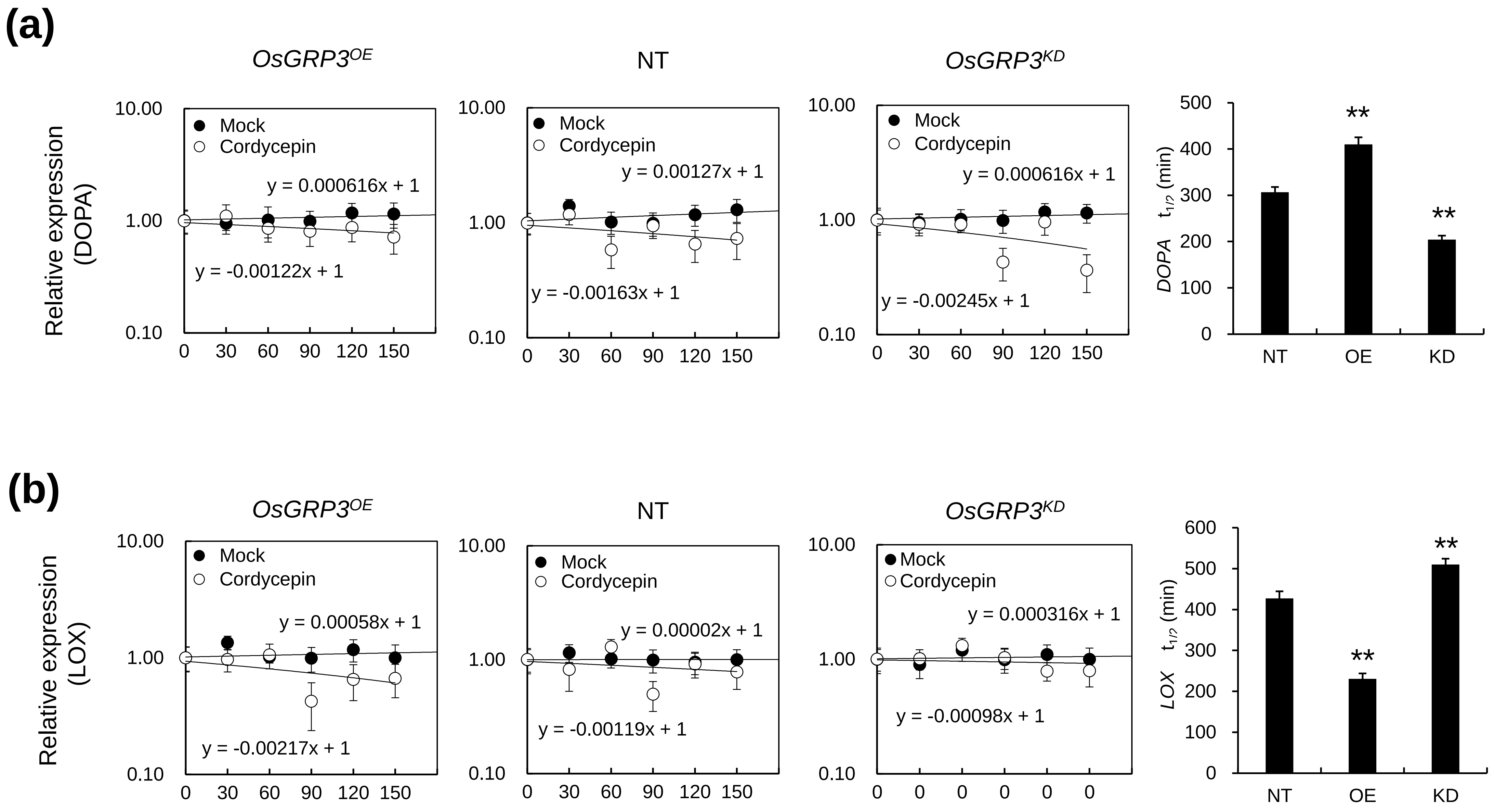


**Figure S9. Effects of *OsGRP3* on stability of *DOPA* and *LOX* transcripts. (a *and* b)** Stability of *DOPA* **(a)** and *Mt1d* **(b)** transcripts was analyzed under drought conditions. Two-week-old non-transgenic (NT), *OsGRP3* overexpression (*OsGRP3*$^{OE}$) and RNAi-mediated *OsGRP3* suppressing (*OsGRP3*$^{KD}$) transgenic plants were pre-treated with distilled water (closed circle) or 1 mM cordycepin for 30 minutes (open circle). Plants were then exposed to drought stress by air-drying and harvested every 30 minutes after the treatments. Total RNAs extracted from the harvested samples were applied for qRT-PCR analysis. Data represent mean value of three replicates. Regression curves and were plotted using sigma plot software (https://systatsoftware.com/). Half-life of transcripts were calculated based on regression curve. Asterisks indicate statistically significant difference compared with NT as analyzed by one-way ANOVA followed by t -test, $^{**}P < 0.01$.

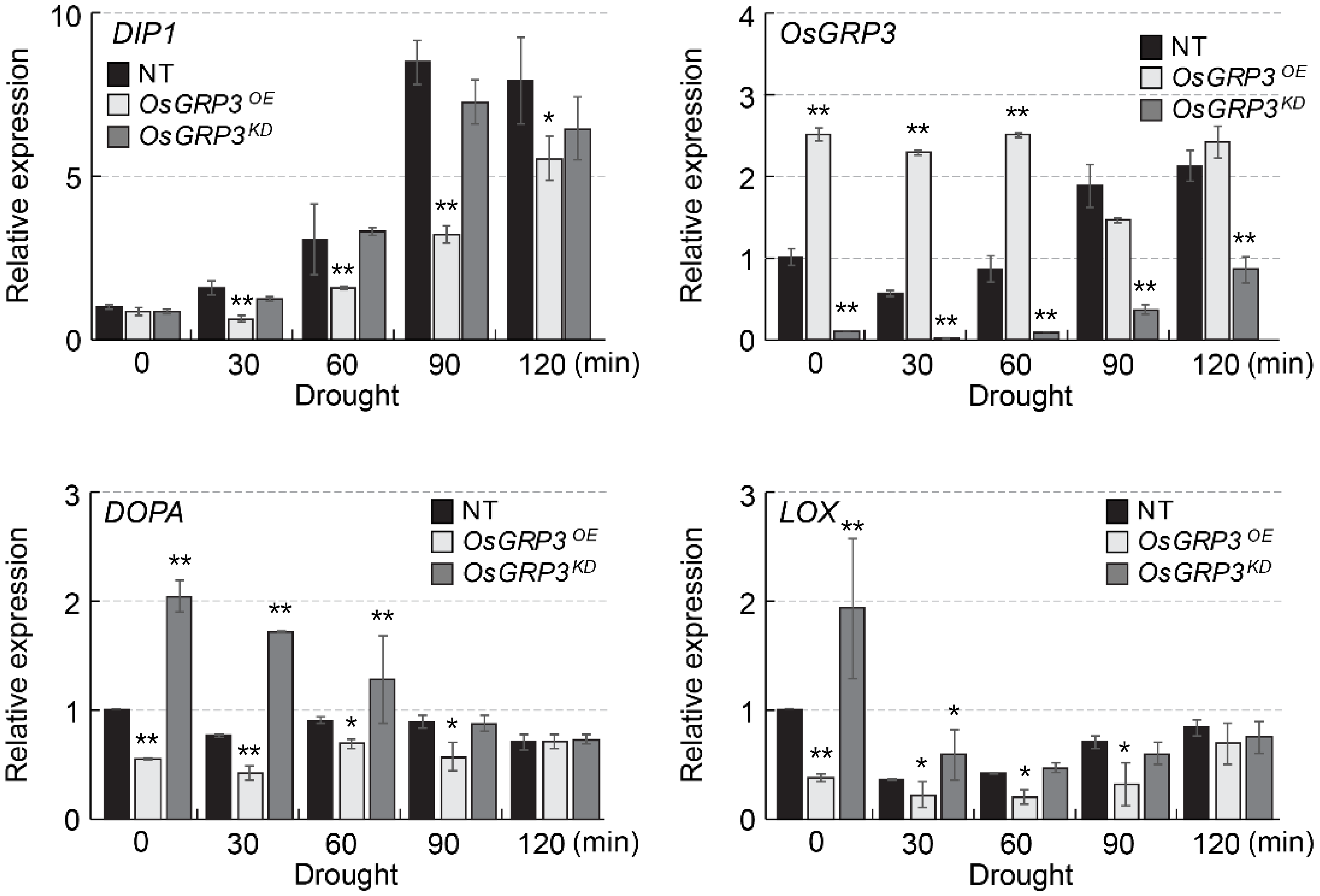


**Figure S10. Expression patterns of *OsGRP3*, *DOPA* and *LOX* under drought conditions.** Two-week-old non-transgenic (NT), *OsGRP3* overexpressing (*OsGRP3*$^{OE}$) and RNAi-mediated *OsGRP3* suppressing (*OsGRP3*$^{KD}$) transgenic plants were exposed to drought stress by air-drying and harvested at indicated time points. Total RNAs isolated from harvested leaves were analyzed by qRP-PCR. Rice *DIP1* was used as a positive control for drought treatments. Rice *UBIQUITIN* (*OsUbi*) was used as internal control for normalization. Data represent mean value ± standard deviation (SD) (n=3). Asterisks indicate statistically significant difference compared with NT as analyzed by one-way ANOVA followed by t -test: *P $< 0.05$, **P $< 0.01$.
